# A polo-like kinase inhibitor identified by computational repositioning attenuates pulmonary fibrosis

**DOI:** 10.1186/s12931-023-02446-x

**Published:** 2023-06-02

**Authors:** Takeshi Imakura, Seidai Sato, Kazuya Koyama, Hirohisa Ogawa, Takahiro Niimura, Kojin Murakami, Yuya Yamashita, Keiko Haji, Nobuhito Naito, Kozo Kagawa, Hiroshi Kawano, Yoshito Zamami, Keisuke Ishizawa, Yasuhiko Nishioka

**Affiliations:** 1 Department of Respiratory Medicine and Rheumatology, Graduate School of Biomedical Sciences, Tokushima University, 3-18-15 Kuramoto-cho, Tokushima, 770-8503 Japan; 2 Department of Pathology and Laboratory Medicine, Graduate School of Biomedical Sciences, Tokushima University, Tokushima, Japan; 3 Department of Clinical Pharmacology and Therapeutics, Graduate School of Biomedical Sciences, Tokushima University, Tokushima, Japan; 4 Department of Pharmacy, Okayama University Hospital, Okayama, Japan

**Keywords:** Polo-like kinase, Pulmonary fibrosis, In silico screening

## Abstract

**Background:**

Idiopathic pulmonary fibrosis (IPF) is a fatal fibrotic lung disease with few effective therapeutic options. Recently, drug repositioning, which involves identifying novel therapeutic potentials for existing drugs, has been popularized as a new approach for the development of novel therapeutic reagents. However, this approach has not yet been fully utilized in the field of pulmonary fibrosis.

**Methods:**

The present study identified novel therapeutic options for pulmonary fibrosis using a systematic computational approach for drug repositioning based on integration of public gene expression signatures of drug and diseases (in silico screening approach).

**Results:**

Among the top compounds predicted to be therapeutic for IPF by the in silico approach, we selected BI2536, a polo-like kinase (PLK) 1/2 inhibitor, as a candidate for treating pulmonary fibrosis using an in silico analysis. However, BI2536 accelerated mortality and weight loss rate in an experimental mouse model of pulmonary fibrosis. Because immunofluorescence staining revealed that PLK1 expression was dominant in myofibroblasts while PLK2 expression was dominant in lung epithelial cells, we next focused on the anti-fibrotic effect of the selective PLK1 inhibitor GSK461364. Consequently, GSK461364 attenuated pulmonary fibrosis with acceptable mortality and weight loss in mice.

**Conclusions:**

These findings suggest that targeting PLK1 may be a novel therapeutic approach for pulmonary fibrosis by inhibiting lung fibroblast proliferation without affecting lung epithelial cells. In addition, while in silico screening is useful, it is essential to fully determine the biological activities of candidates by wet-lab validation studies.

**Supplementary Information:**

The online version contains supplementary material available at 10.1186/s12931-023-02446-x.

## Background

Idiopathic pulmonary fibrosis (IPF) is a chronic devastating disease characterized by the proliferation of fibroblasts, excessive accumulation of myofibroblasts, and deposition of extracellular matrix [1] [2]. The prognosis of IPF is poor, with a median survival of about two to four years after the diagnosis [3] [4]. IPF occurs worldwide, and the prevalence of this disease appears to be increasing [5]. Although two antifibtotic drugs, nintedanib and pirfenidone, are currently approved by the FDA for IPF [6], their efficacy is not yet sufficient for patients, as the benefit is limited to inhibiting the decline in the lung function. Therefore, the development of novel drugs is strongly desirable.

The current drug research and development process involves creating a novel reagent, conducting preclinical and clinical studies, and obtaining approval for marketing through the pharmaceutical affairs review process. However, the drug discovery paradigm at present not only is generally costly and time-consuming but also carries an extremely low success rate of 1 in 20,000 to 30,000 [7].

One way to circumvent this problem is to identify new uses for existing drugs, known as “drug repositioning”. Because existing drugs have known pharmacokinetics and safety profiles and are often approved by regulatory agencies for human use, any newly identified use can be rapidly evaluated in clinical trials, which have a typically short period and are inexpensive to perform [8] [9] [10]. Therefore, drug repositioning is becoming popular as a new method of optimizing the preclinical process of new drug development, saving time and money over the traditional de novo drug discovery process [11].

Recently, large-scale biomedical data and drug databases, such as microarray gene expression signatures and pharmaceutical databases, have also become available to the public along with high-performance computing, enabling the development of computational drug repositioning approaches known as in silico screening [12] [13]. However, very little research has been done on the effective use of drug repositioning in the field of pulmonary fibrosis. Few reports have described the search for new drug candidates using in silico screening [14] [15], and most in existence have simply identified candidate drugs, with no validation of their actual efficacy using in vivo or in vitro studies.

Therefore, in the present study, we not only explored novel therapeutic candidates for pulmonary fibrosis by an in silico screening approach using multi-source data and integrative molecular network bioinformatics but also validated the antifibrotic effect of candidates in an experimental mouse model of pulmonary fibrosis and in vitro study using lung fibroblasts and lung epithelial cells. We consequently found that a polo-like kinase (PLK) inhibitor selected by the combined results of in silico screening and wet-lab validation studies ameliorated pulmonary fibrosis in experimental mouse models of lung fibrosis.

## Methods

The detailed methods are described in the online supplement.

### In silico analyses

The differentially expressed genes (DEGs) between IPF and normal samples were detected from the Gene Expression Omnibus (GEO) database. By inputting DEG data into the L1000 Characteristic Direction Signature Search Engine (L1000CDS2), we searched for a therapeutic candidate of IPF.

### Isolation of primary murine lung epithelial cells

Murine lung epithelial cell purification was performed using negative depletion with CD45 MicroBeads and positive selection for the epithelial-cell adhesion molecule (Ep-CAM), as previously described [16].

### Proliferation assays

Murine primary lung fibroblasts were seeded onto 96-well plates and cultured with various concentrations of BI2536 or GSK461364 in the presence of fibroblast growth factor-2 (FGF-2) (30 ng/ml) or platelet-derived growth factor (PDGF) (100 ng/ml) for 48 h. Murine lung epithelial cells were seeded onto 96-well plates and cultured with various concentrations of BI2536 or GSK461364 in the presence of FGF-2 (30 ng/ml) for 48 h. One microcurie per well of [^3^H] thymidine deoxyribose was pulsed for the final 24 h, and the incorporation of [^3^H] thymidine deoxyribose was measured using a liquid scintillation counter [17].

### Immunoblot analyses

Cell extracts of murine fibroblasts and lung epithelial cells were lysed and used for immunoblotting, as previously described [18].

### Bleomycin-induced pulmonary fibrosis in mice

Eight-week-old C57BL/6 mice were purchased from Charles River Japan (Kanagawa, Japan). Mice received a single intra-tracheal instillation of bleomycin (3 mg/kg) on day 0. BI2536 (5 mg/kg, 10 mg/kg or 20 mg/kg) was administered twice a week from day 0 until day 21. GSK461364 (5 mg/kg) was administered twice a week from day 0 until day 21. Lung tissues were analyzed on Day 21.

### Histopathology

Right lung tissues were harvested, fixed in 10% formalin, and embedded in paraffin. Three-micrometer-thick sections were stained with hematoxylin and eosin (H&E) or Azan Mallory. In the quantitative analysis, a numeric fibrotic scale (Ashcroft score) was used.

### Hydroxyproline colorimetric assay

The hydroxyproline contents of the bleomycin-treated lungs were measured using a hydroxyproline colorimetric assay kit (BioVision).

### Bronchoalveolar lavage

Bronchoalveolar lavage (BAL) was performed with saline (1 mL) using a soft cannula. After counting the number of cells in the BAL fluid (BALF), cells were stained with Diff-Quick for cell classification.

### Immunofluorescence staining

Paraffin-embedded lung sections were stained with primary antibodies at 4°C overnight and subsequently stained with fluorescence-conjugated secondary antibodies and 4’, 6-diamidino-2-phenylindole at room temperature for 1 h. Fluorescence images were captured with a confocal laser scanning microscope.

### Quantitative real-time polymerase chain reaction

Quantitative real-time polymerase chain (PCR) was performed as previously described [19].

### Statistical analyses

The significance of differences was analyzed using an unpaired *t*-test for comparisons between two groups, or a one-way analysis of variance followed by Dunnett’s test for comparisons between more than two groups. *P* values of < 0.05 were considered to indicate statistical significance. These statistical analyses were performed with the GraphPad Prism software program (Ver. 5.01; GraphPad Software Inc., Prism Software, Irvine, CA, USA).

## Results

### We identified candidate drugs to attenuate pulmonary fibrosis

To explore the novel therapeutic candidate of pulmonary fibrosis, we applied a practical in silico screening approach using multi-sources data information and integrative molecular network bioinformatics. First, we obtained the microarray data of IPF from the GEO database and compared microarray data from IPF patients (n = 26) with those from normal patients (n = 9) registered with the GEO as GSE5774 in order to detect DEGs (defined as an adjusted *P* value of < 0.05 and |log_2_FC| > 0.585). As a result, we detected 833 DEGs.

**Fig. 1:**
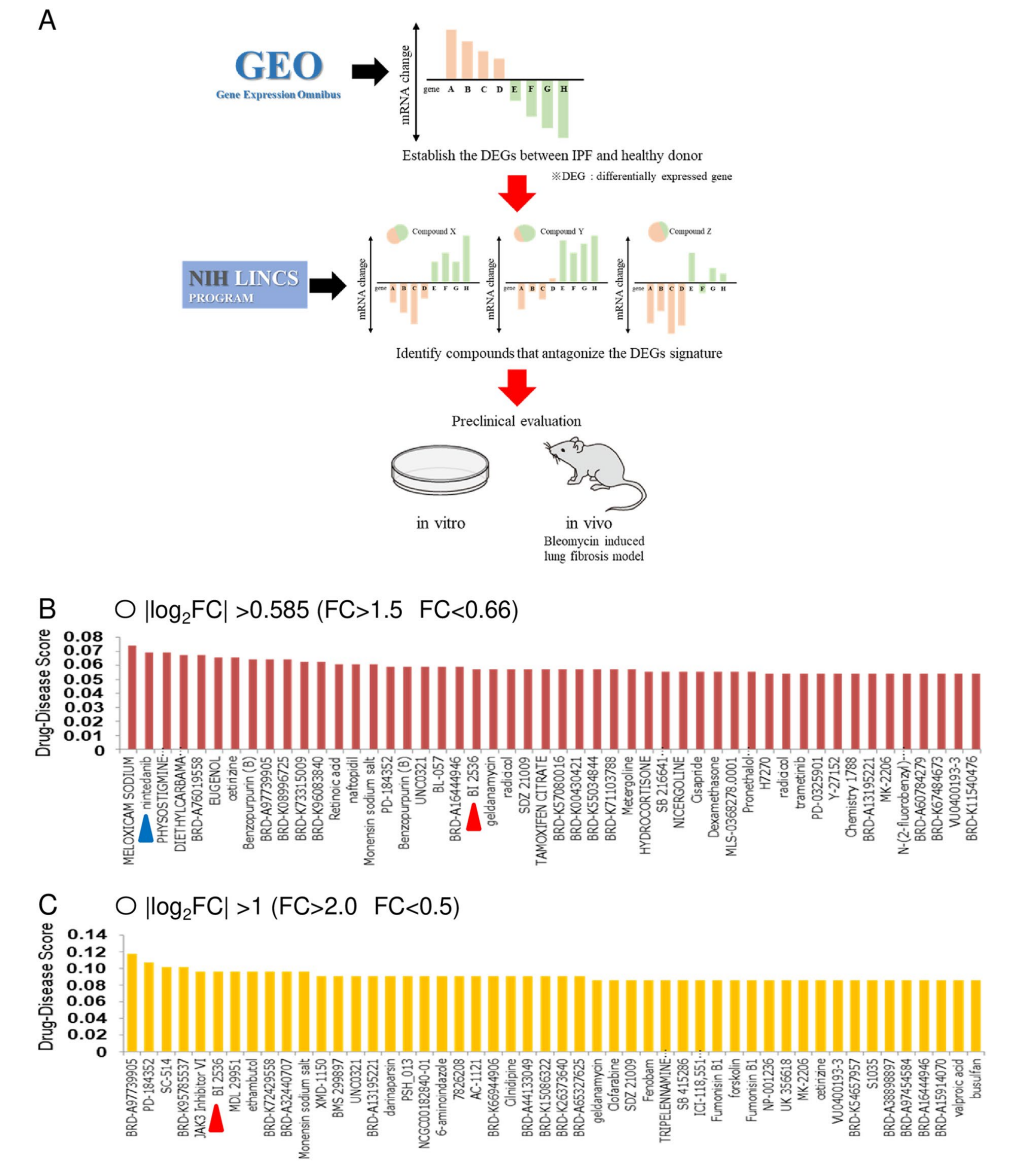
Computational repositioning identifies a polo-like kinase 1/2 inhibitor as a candidate drug for pulmonary fibrosis. (A) Schematic of the in-silico approach used to identify candidate drugs that attenuate pulmonary fibrosis. Microarray data of lung tissue of patients with idiopathic pulmonary fibrosis (IPF) were extracted from the Gene Expression Omnibus database. By comparing them with microarray data from healthy donors, the differentially expressed genes (DEGs) were established. Therapeutic candidate drugs that antagonize the DEG signature were identified using drug signatures obtained from the Library of integrated Network-based Cellular Signatures. (B) Drug-disease score represents the agreement between the input genetic variation data and the candidate drug’s genetic variation data. The top 50 candidate drugs with the highest drug-disease scores when the fold-change (FC) of DEGs was |log_2_FC| > 0.585 (> 1.5 and < 0.66) are shown. (C) The top 50 candidate drugs with the highest drug-disease scores when FC of DEGs is |log_2_FC| > 1 (> 2.0 and < 0.5) are shown. The blue triangle points toward nintedanib, which was approved for the treatment of IPF. The red triangle points toward BI2536, a polo-like kinase 1/2 inhibitor that was selected for a therapeutic candidate of IPF

We next searched for therapeutic candidates for IPF by inputting DEGs into the L1000CDS^2^ (Fig. 1A). When the condition was |log_2_FC| > 0.585, nintedanib, which is currently approved for IPF treatment, was listed as the second-best candidate (Fig. 1B). We selected BI2536, a polo-like kinase 1/2 inhibitor, as a therapeutic candidate drug. PLK is serine/threonine kinase that regulates the cell cycle [20]. We selected BI2536 because it had no previous reports concerning any anti-fibrotic effects, was ready-made and available, and was ranked higher based on the strict criterion of |log_2_FC| > 1 (Fig. 1C).

### The PLK1/2 inhibitor accelerated the body weight loss and increased the mortality of bleomycin-treated mice

**Fig. 2:**
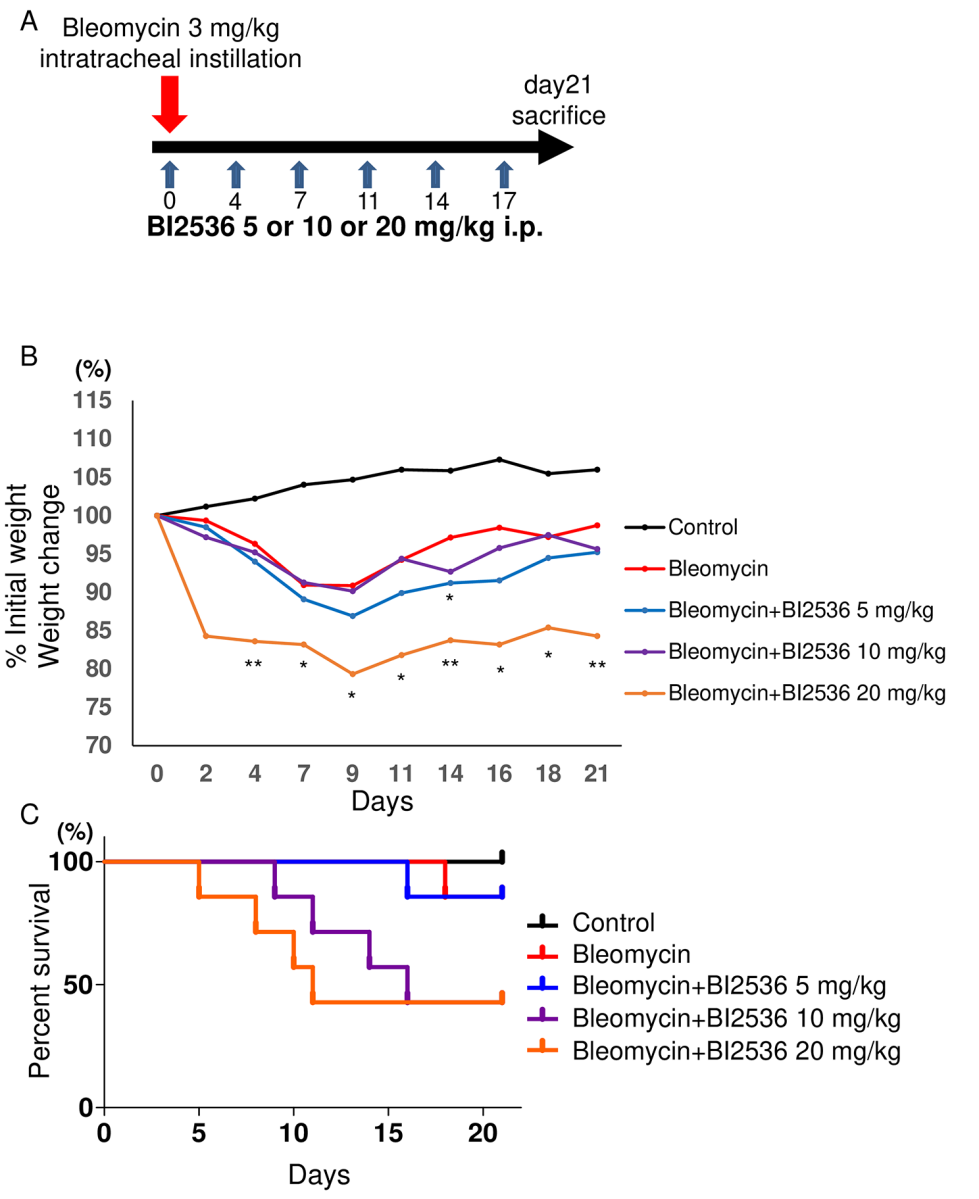
BI2536 accelerated the body weight loss and increased the mortality of bleomycin-treated mice. (A) C57BL/6 mice received bleomycin (3 mg/kg, intratracheal instillation) on day 0. BI2536 (injected intraperitoneally at a dose of 5, 10 or 20 mg/kg) or vehicle was administered twice a week. Analyses were performed on day 21. The change in the body weight of each group (B) and a Kaplan-Meier plot showing the survival in each group (C) (n = 3, control group; n = 7, bleomycin-alone group; n = 7, bleomycin + BI2536 5 mg/kg group; n = 7, bleomycin + BI2536 10 mg/kg group; n = 7, bleomycin + BI2536 20 mg/kg group). The results of body weight on each day in the bleomycin + BI2536 5, 10 and 20 mg/kg group were analyzed using a one-way analysis of variance followed by Dunnett’s multiple comparison test. * *P =* 0.01–0.05; ** *P* = 0.001–0.01, which are versus the value in the group treated with bleomycin only

To investigate whether or not BI2536 had anti-fibrotic effects, we induced pulmonary fibrosis in mouse lungs with bleomycin. C57BL/6 mice received a single intra-tracheal instillation of bleomycin (3 mg/kg) on day 0. BI2536 (5 mg/kg, 10 mg/kg or 20 mg/kg) or vehicle was then administered via intraperitoneal instillation twice a week until day 21. Lung tissues were harvested and analyzed on day 21 (Fig. 2A). The weight of mice in the bleomycin-alone group gradually started to decrease on day 2 (Fig. 2B). Surprisingly, in the bleomycin + BI2536 (20 mg/kg) group, the body weight started to decrease soon after the intratracheal instillation of bleomycin. The rate of reduction was higher than in the bleomycin-alone group, and a significant difference was found on days 4, 7, 9, 11, 14, 16, 18, and 21 (Fig. 2B). Along with the decrease in the body weight, the survival rate in the bleomycin + BI2536 (20 mg/kg) group began to decline from an early period, and as a result the total survival rate was lower than that in the bleomycin-alone group (Fig. 2C). The survival rate in the bleomycin + BI2536 (10 mg/kg) group was also lower than that in the bleomycin-alone group (Fig. 2C).

However, by the continuous administration of BI2536 at doses of 10 mg/kg or 20 mg/kg, the number of fibrotic lesions in the lungs of bleomycin-treated mice was reduced (Figure E1A). A quantitative histological analysis showed that the Ashcroft fibrotic score was significantly lower in mice treated with bleomycin + BI2536 than in those treated with bleomycin alone except bleomycin + BI2536 (5 mg/kg) group (Figure E1B).

These results suggest that the administration of a PLK1/2 inhibitor at sufficient doses improves pulmonary fibrosis but worsens the rate of weight loss and survival. On the other hand, dose reduction or late administration of PLK1/2 inhibitors improved weight loss and survival of mice, but had insufficient antifibrotic effects.

### PLK1 expression was dominant in myofibroblasts, whereas PLK2 expression was dominant in lung epithelial cells

To examine why the PLK1/2 inhibitor BI2536 increased the mortality of bleomycin-treated mice, we next focused on the localization of PLKs. Although PLKs consists of one family and includes five members (PLK1-5), the localization of each PLK in the lung has not been fully studied [20]. Among the vertebrate PLK family members, PLK1 has been most extensively studied, and it along with PLK2 have been reported to be involved in cell proliferation in the G2-M phase and G1 phase, respectively [20].

**Fig. 3:**
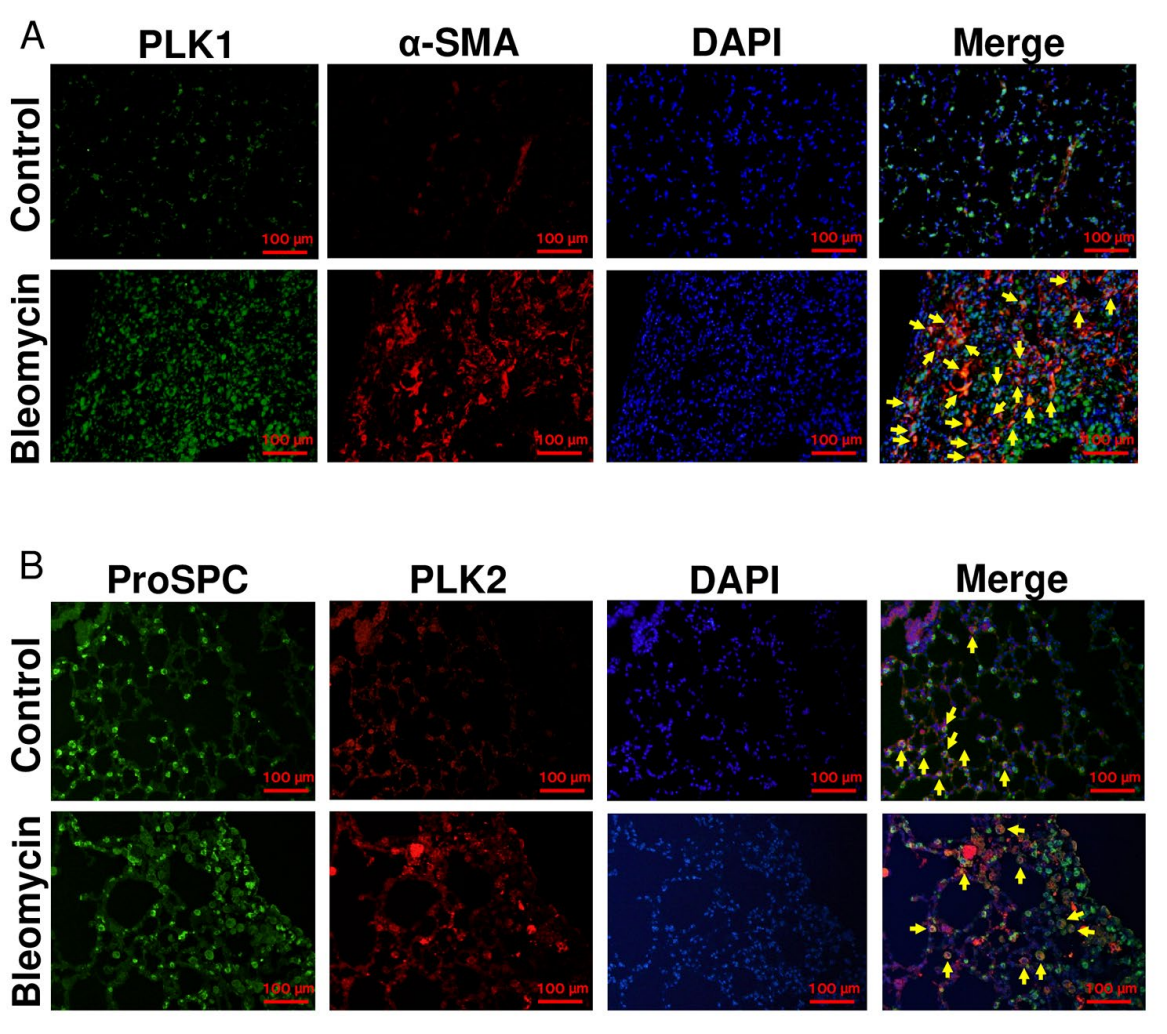
PLK1 expression in myofibroblasts and PLK2 expression in lung epithelial cells. C57BL/6 mice received bleomycin (3 mg/kg, intratracheal instillation) on day 0. The lung tissue was harvested on day 21. (A-B) Paraffin-embedded lung sections were stained with an anti-PLK1 antibody (green) and anti-α-SMA antibody (red) or with an anti-pro-SPC antibody (green) and anti-PLK2 antibody (red). Representative images of immunofluorescence staining in control group or bleomycin-treated group are shown. Yellow arrows indicate cells double-positive for PLK1 and α-SMA (A), for PLK2 and pro-SPC (B). Scale bars, 100 mm

Immunofluorescence staining revealed that the number of PLK1^+^ cells was increased in fibrotic regions (Fig. 3A, E2A). In particular, PLK1^+^ cells were co-localized with alpha smooth muscle actin (α-SMA) ^+^ cells (Fig. 3A) but not with pro-surfactant Protein C (pro-SPC) ^+^ cells (Figure E2A). Conversely, the number of PLK2^+^ cells was decreased in fibrotic regions (Fig. 3B, E2B). Immunofluorescence staining revealed that PLK2^+^ cells were co-localized with pro-SPC^+^ cells (Fig. 3B) but not with α-SMA^+^ cells (Figure E2B).

We extracted mRNA from CD45^−^ epithelial-cell adhesion molecule (Ep-CAM)^−^ cells, a fibroblast-enriched population, and CD45^−^Ep-CAM^+^ cells and performed quantitative polymerase chain reaction (PCR) for *Plk1* and *Plk2*. The mRNA expression of *Plk1* was significantly higher in CD45^−^Ep-CAM^−^ cells than in CD45^−^Ep-CAM^+^ (Figure E2C), but the expression of *Plk*2 was higher in CD45^−^Ep-CAM^+^ than in CD45^−^Ep-CAM^−^ cells (Figure E2D).

Hence, in the comparison between these two types of cells, these results suggest that PLK1 is preferentially expressed in myofibroblasts, while PLK2 is preferentially expressed in lung epithelial cells.

### A selective PLK1 inhibitor attenuated pulmonary fibrosis with acceptable mortality and weight loss

In developing new anti-fibrotic drugs for pulmonary fibrosis, especially when targeting factors related to proliferation, such as PLKs, it is desirable for an agent to be effective on fibroblasts but have less effect on lung epithelial cells. Therefore, we hypothesized that the inhibition of PLK1 alone would have an anti-fibrotic effect without suppressing the recovery of lung epithelial cells from injury and examined the anti-fibrotic effect of GSK461364, a selective PLK1 inhibitor, in a mouse model of bleomycin-induced pulmonary fibrosis.

**Fig. 4:**
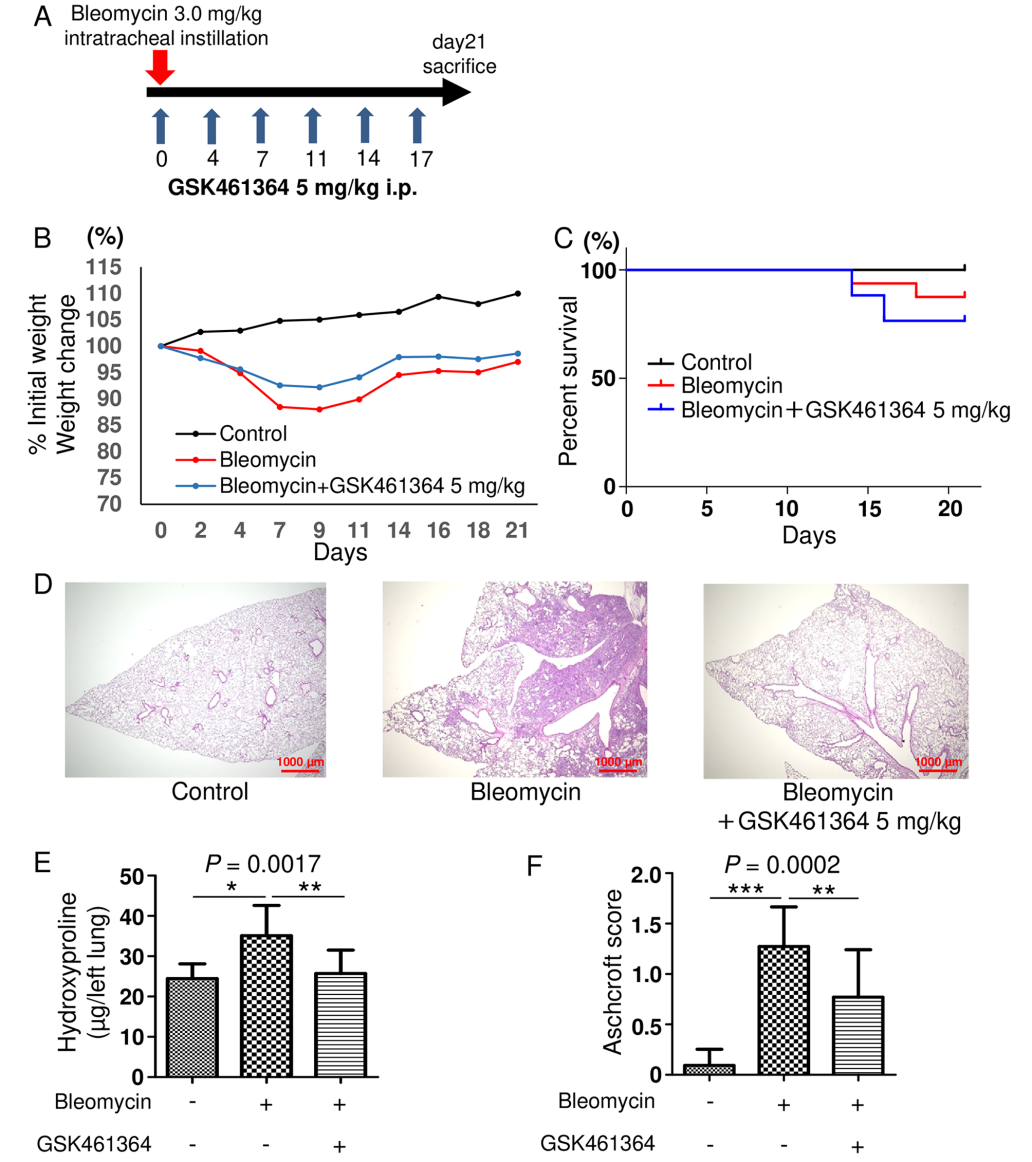
GSK461364 attenuated pulmonary fibrosis with acceptable mortality and weight loss. (A) C57BL/6 mice received bleomycin (3 mg/kg, intratracheal instillation) on day 0. GSK461364 (injected intraperitoneally at a dose of 5 mg/kg) or vehicle was administered twice a week. Analyses were performed on day 21. The change in the body weight of each group (B) and a Kaplan-Meier plot showing the survival in each group (C) (n = 3, control group; n = 16, bleomycin-alone group; n = 17, bleomycin + GSK461364 5 mg/kg group). (D) A histological examination was performed with hematoxylin and eosin staining. Scale bars, 1000 μm. (E) The fibrotic changes in the lungs were quantified with a numerical fibrotic score (Ashcroft score) histopathologically (n = 3, control group; n = 14, bleomycin-alone group; n = 13, bleomycin + GSK461364 5 mg/kg group). (F) The collagen content in the left-lung lobe was measured using a hydroxyproline colorimetric assay (n = 3, control group; n = 14, bleomycin-alone group; n = 13, bleomycin + GSK461364 5 mg/kg group). Data were analyzed using a one-way analysis of variance followed by Dunnett’s multiple comparison test and were displayed as the mean ± SD. For all graphs: * *P =* 0.01–0.05; ** *P* = 0.001–0.01; *** *P* < 0.0001. The *P* values of each experiment are shown above each figure

After mice received intratracheal instillation of bleomycin, GSK461364 (5 mg/kg) was administered by intraperitoneal instillation twice a week until day 21. Lung tissues were then harvested and analyzed on day 21 (Fig. 4A). In contrast to the experiments with BI2536, there was no marked difference in the rate of weight loss or survival between the bleomycin-alone and bleomycin + GSK461364 groups (Fig. 4B C). Furthermore, GSK461364 attenuated bleomycin-induced lung fibrosis (Fig. 4D) and reduced the collagen content in the lungs (Fig. 4E). A quantitative histological analysis also showed that the Ashcroft fibrotic score was significantly lower in mice treated with bleomycin + GSK461364 than in those treated with bleomycin alone (Fig. 4F). These results suggest that a selective PLK1 inhibitor attenuated pulmonary fibrosis without worsening the rate of weight loss or survival.

### Selective PLK1 inhibition does not affect the infiltration of inflammatory cells into the lungs

To investigate why selective PLK1 inhibition ameliorated lung fibrosis in bleomycin-treated mice, we next examined whether or not GSK461364 treatment affected the number of inflammatory cells and lymphocyte fractions in BALF. The BALF was harvested from mice in each group on day 21. A BALF analysis showed no marked differences in the cell count, macrophage percentage, lymphocyte percentage, or neutrophil percentage between the bleomycin-alone group and the bleomycin + GSK461364 group (Figure E3A-E3D).

### Selective PLK1 inhibitor does not inhibit the proliferation of lung epithelial cells in vivo

**Fig. 5:**
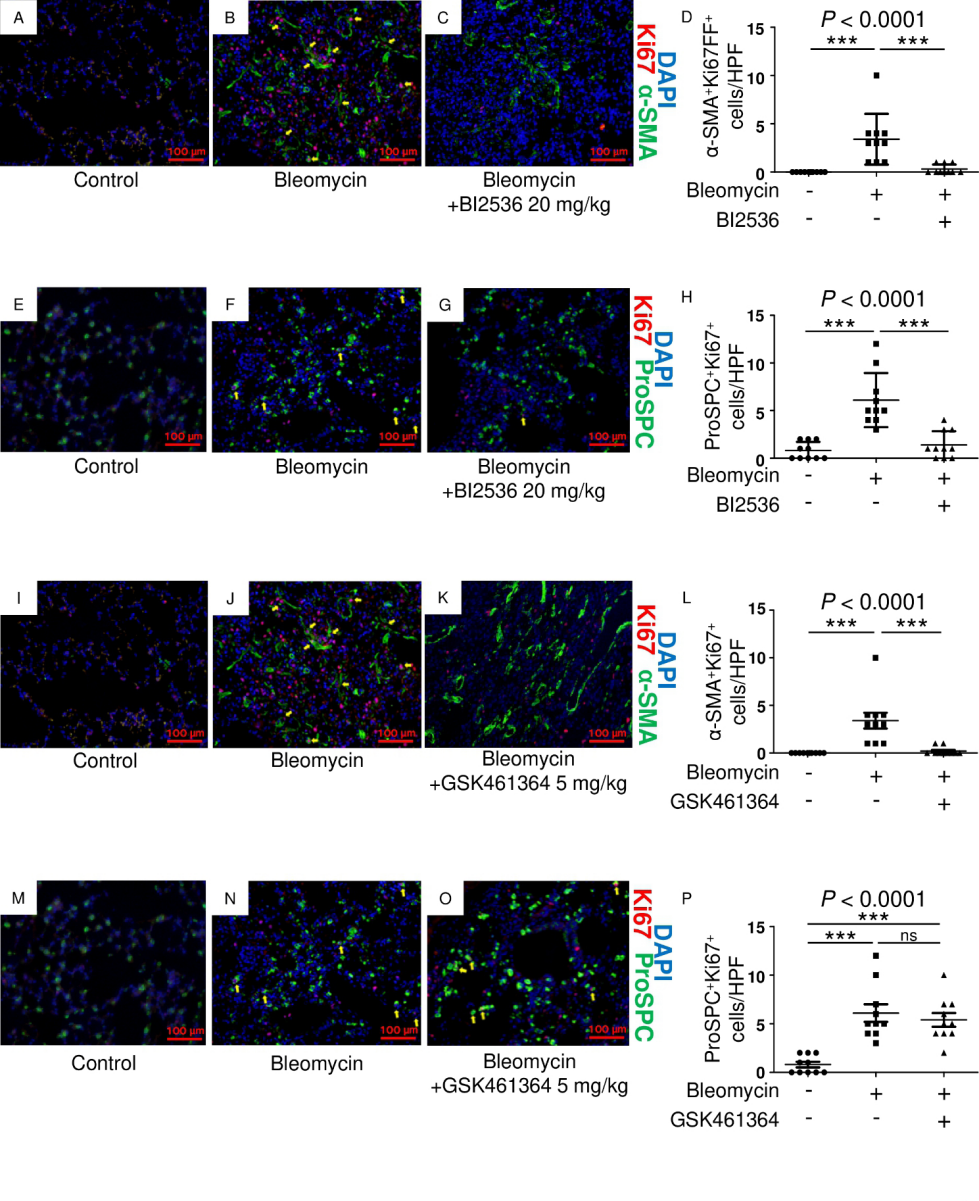
GSK461364 suppresses the proliferation of fibroblasts but not that of lung epithelial cells in vivo. C57BL/6 mice received bleomycin (3 mg/kg, intratracheal instillation) on day 0. BI2536 (20 mg/kg, injected intraperitoneally) or vehicle was administered twice a week. The lung tissue was harvested on day 21. Paraffin-embedded lung sections were stained with an anti-pro-SPC antibody (green) and anti-Ki-67 antibody (red) or with an anti-α-SMA antibody (green) and anti-Ki-67 antibody (red). Representative images of immunofluorescence staining in each group are shown (A, E: control group; B, F: bleomycin-alone group; C, G: bleomycin and BI2536 20 mg/kg group). Yellow arrows indicate cells double-positive for α-SMA and Ki-67 (A-C), for pro-SPC and Ki-67 (E-G). Scale bars, 100 μm. (D) The α-SMA^+^ Ki-67^+^cells were counted in 10 random fields using 3 lung sections. Data are displayed as dot plots and the mean. (H) The pro-SPC^+^Ki-67^+^cells were counted in 10 random fields using 3 lung sections. Data are displayed as dot plots and the mean. C57BL/6 mice received bleomycin (3 mg/kg, intratracheal instillation) on day 0. GSK461364 (5 mg/kg, injected intraperitoneally) or vehicle was administered twice a week. The lung tissue was harvested on day 21. Paraffin-embedded lung sections were stained with an anti-pro-SPC antibody (green) and anti-Ki-67 antibody (red) or with an anti-α-SMA antibody (green) and anti-Ki-67 antibody (red). Representative images of immunofluorescence staining in each group are shown (I, M: control group; J, N: bleomycin-alone group; K, O: bleomycin and GSK461364 5 mg/kg group). Yellow arrows indicate cells double-positive for α-SMA and Ki-67 (I-K), for pro-SPC and Ki-67 (M-O). Scale bars, 100 μm. Data were analyzed using a one-way analysis of variance followed by Dunnett’s multiple comparison test and displayed as dot plots and the mean ± SD. For all graphs: NS = not significant; *** *P* < 0.0001. The *P* values of each experiment are shown above each figure

To examine the effects of BI2536 on lung fibroblasts or epithelial cells, we harvested lung tissue from mice administered bleomycin or bleomycin and BI2536 20 mg/kg on day 21. Paraffin-embedded lung sections were stained for the fibroblast marker α-SMA or the epithelial cell marker pro-SPC and the proliferation marker Ki-67. Immunohistochemical staining revealed that the number of α-SMA^+^Ki-67^+^ cells in the bleomycin + BI2536 group was significantly lower than that in the bleomycin-alone group (Fig. 5A and D). Furthermore, the number of pro-SPC^+^Ki-67^+^ cells in the bleomycin + BI2536 group was also significantly lower than in the bleomycin-alone group (Fig. 5E H).

In contrast, although the number of α-SMA^+^Ki-67^+^ cells in the bleomycin + GSK461364 group was significantly lower than that in the bleomycin-alone group (Fig. 5I L), the number of pro-SPC^+^Ki-67^+^ cells was not decreased by GSK461364 (Fig. 5M and P).

Taken together, these data suggest that a PLK1/2 inhibitor inhibited the proliferation of both lung fibroblasts and epithelial cells, while a selective PLK1 inhibitor inhibited the proliferation of lung fibroblasts only.

### Growth factors upregulates PLK1 in lung fibroblasts and PLK2 in lung epithelial cells

The fact that growth factors play a pivotal role for the fibrotic process by inducing fibroblast proliferation is now widely accepted. Indeed, numerous reports suggest that FGF and PDGF are involved in the pathogenesis of pulmonary fibrosis [21–26][17]. Therefore, we next examined whether or not these growth factors affect the expression of PLK, a regulator of the cell cycle.

**Fig. 6:**
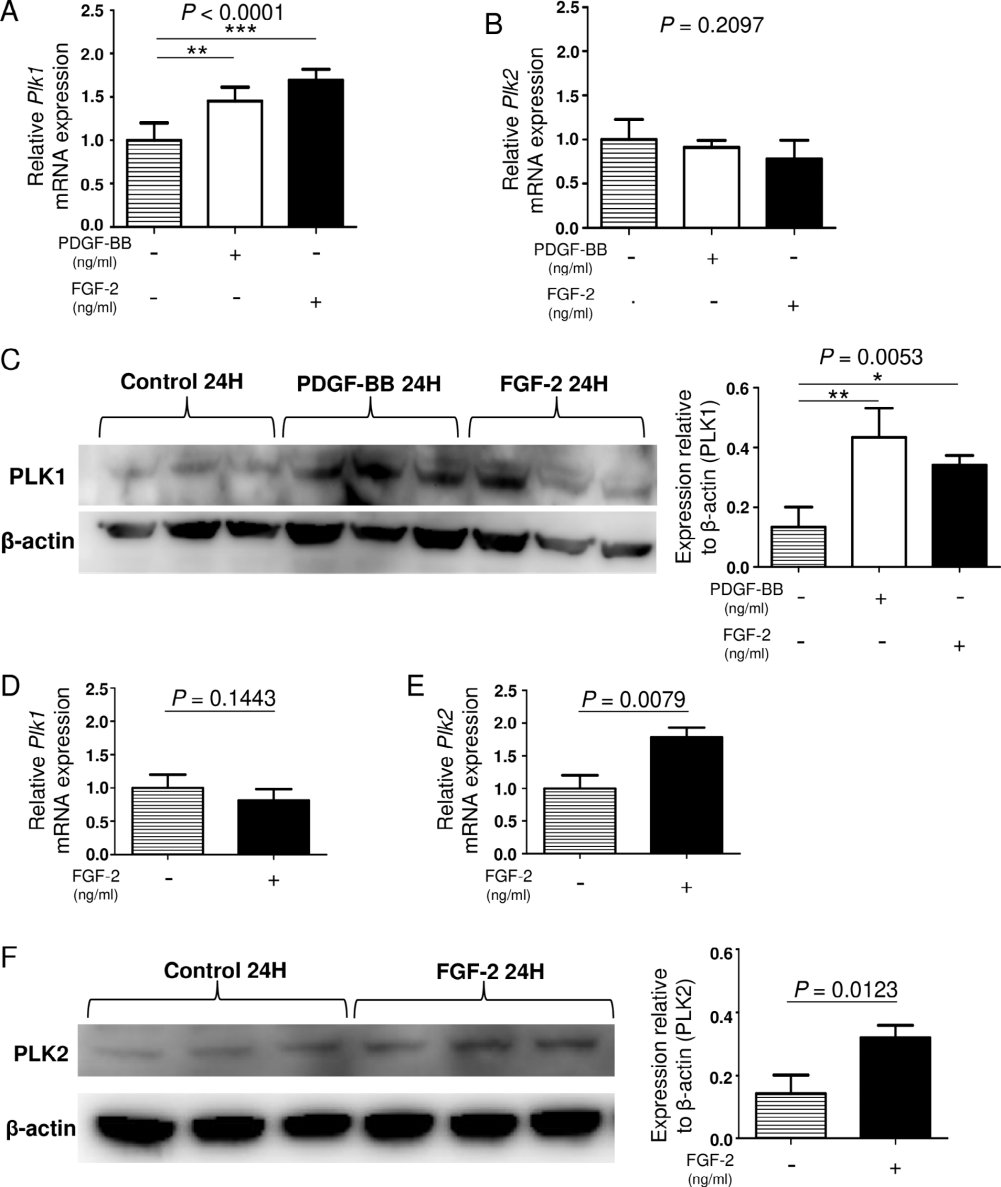
The expression of PLKs in response to growth factors differs among cell types. (A-B) The mRNA expression of *Plk1* or *Plk2* in cultured murine primary lung fibroblasts stimulated with PDGF-BB 100 ng/ml or FGF-2 30 ng/ml for 24 h was analyzed by quantitative PCR (n = 5 in control group; n = 5 in PDGF-BB group; n = 5 in FGF-2 group). (C) The protein expression of PLK1 in cultured primary lung fibroblasts stimulated with PDGF-BB 100 ng/ml or FGF-2 30 ng/ml for 24 h was analyzed by Western blotting (n = 3 in control group; n = 3 in PDGF-BB group; n = 3 in FGF-2 group). Data were analyzed using a one-way analysis of variance followed by Dunnett’s multiple comparison test and displayed as the mean ± SD. For all graphs: * *P =* 0.01–0.05; ** *P* = 0.001–0.01; *** *P* < 0.0001. The *P* values of each experiment are shown above each figure. (D-E) The mRNA expression of *Plk1* or *Plk2* in cultured murine alveolar epithelial type 2-like (LA4) cells stimulated with FGF-2 30 ng/ml for 24 h was analyzed by quantitative PCR (n = 5 in control group; n = 5 in FGF-2 group). (F) The protein expression of PLK2 in cultured LA4 cells stimulated with FGF-2 30 ng/ml for 24 h was analyzed by Western blotting (n = 3 in control group; n = 3 in FGF-2 group). Data were analyzed using unpaired *t*-test and displayed as the mean ± SD. The *P* values of each experiment are shown above each figure

Because PDGFR is expressed on lung fibroblasts but not on lung epithelial cells, the effect of PDGF was examined only on lung fibroblasts [27]. At the mRNA level, the stimulation with PDGF-BB or FGF-2 significantly upregulated the expression of *Plk1* in murine primary lung fibroblasts (Fig. 6A). In contrast, the expression of *Plk2* was not changed by PDGF-BB or FGF-2 stimulation (Fig. 6B). Similarly, the protein level of PLK1 in murine primary lung fibroblasts was also significantly upregulated by PDGF-BB or FGF-2 stimulation (Fig. 6C).

Conversely, murine lung epithelial cells stimulated with FGF-2 significantly upregulated the mRNA expression of *Plk2* but not *Plk1* (Fig. 6D and E). The PLK2 protein level was also significantly upregulated by FGF-2 stimulation (Fig. 6F).

These data suggest that the stimulation with growth factors induce PLK1 expression in lung fibroblasts and PLK2 in lung epithelial cells.

### PLK1/2 inhibition suppresses both lung fibroblast and epithelial cell proliferation, whereas PLK1 inhibition inhibits only lung fibroblast proliferation

**Fig. 7:**
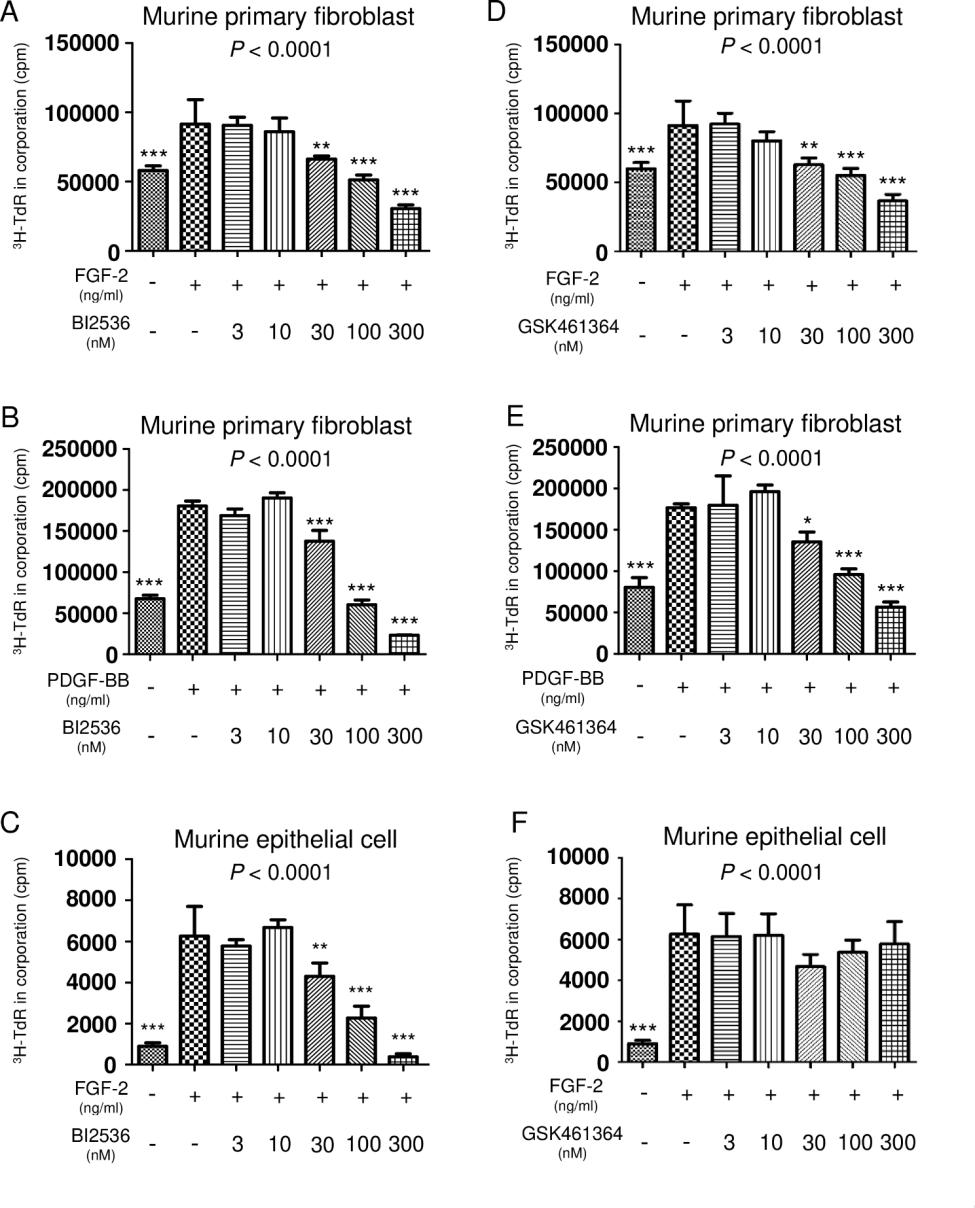
GSK461364 doesn’t inhibit the growth of lung epithelial cells. (A-C) The proliferation of murine primary fibroblasts or LA4 cells cultured in BI2536 stimulated with FGF-2 30 ng/ml or PDGF-BB 100 ng/ml was measured by a ^3^H thymidine incorporation assay. The primary fibroblasts or cultured LA4 cells cultured with 3 to 300 nM of BI2536 for 72 h. ^3^H-TdR was pulsed for the final 18 h, and the incorporation of ^3^H-TdR was measured. (D-F) The proliferation of murine primary fibroblasts or cultured LA4 cells cultured in GSK461364 stimulated with FGF-2 30 ng/ml or PDGF-BB 100 ng/ml was measured by a ^3^H thymidine incorporation assay. The proliferation of murine primary fibroblasts or cultured LA4 cells cultured with 3 nM to 300 nM of GSK461364 for 72 h. ^3^H-TdR was pulsed for the final 18 h, and the incorporation of ^3^H-TdR was measured. Data were analyzed using a one-way analysis of variance followed by Dunnett’s multiple comparison test and displayed as the mean ± SD. For the graph (A), (C), (D), (F) compared with the FGF-2 30 ng/ml group without drug administration: ** *P* = 0.001–0.01; *** *P* < 0.0001. The *P* values of each experiment are shown above each figure. For the graph (B) (E) compared with the PDGF-BB 100 ng/ml group without drug administration: ** *P* = 0.001–0.01; *** *P* < 0.0001. The *P* values of each experiment are shown above each figure

We next examined whether or not PLK inhibition affect the biological responses of cells to growth factors using a ^3^H-TdR incorporation assay. FGF-2 or PDGF-BB significantly upregulated the proliferation of murine primary lung fibroblasts and murine lung epithelial cells (Fig. 7A and E). Proliferation of murine primary lung fibroblasts induced by FGF-2 or PDGF was significantly suppressed by BI2536 or GSK461364 administration at ≥ 30 nM (Fig. 7A, B, D and E). Conversely, murine lung epithelial cell proliferation induced by FGF-2 was significantly suppressed by BI2536 administration at ≥ 30 nM (Fig. 7C) but not by GSK461364 (Fig. 7F).

Taken together, these data suggest that PLK1/2 inhibitors suppressed the proliferation of both lung fibroblasts and epithelial cells, while a selective PLK1 inhibitor suppressed lung fibroblast proliferation but not lung epithelial cell proliferation.

### BI2536 downregulates the mRNA expression of col1a1

Based on the result so far, both BI2536 and GSK461364 seemed to have anti-fibrotic effects. However, only BI2536 (not GSK461364) was selected by our in silico analysis. To determine the reason for this discrepancy, we re-examined genes that were suppressed or upregulated by BI2536 in the data that had been initially analyzed by the drug discovery tool L1000CDS2 to select BI2536 as a therapeutic candidate of IPF (Figure E4A). As a result, BI2536 was found to suppress collagen-related genes, such as *COL1A1*, *COL1A2*, *COL3A1*, *COL5A1*, *COL5A2*, and *COL6A1* (Figure E4A), which was assumed to be the main reason that BI2536 was selected in the in silico analysis. Therefore, we next tried to confirm these inhibitory effects for collage-related genes using quantitative PCR.

The mRNA expression of *Col1a1* upregulated by TGFβ-1 was significantly inhibited by BI2536 (Figure E4B, C). In contrast, it was not inhibited by GSK461364 (Figure E4D, E). The mRNA expression of *Acta2* was not affected by BI2536 or GSK461364.

Therefore, the reason why GSK461364 was not selected by the in silico analysis was deemed to be due to its lack of an inhibitory effect on pro-fibrotic gene expression.

## Discussion

In this study, we selected the PLK1/2 inhibitor BI2536 as a candidate for the treatment of pulmonary fibrosis using an in silico analysis. However, contrary to expectations, BI2536 increased mortality and weight loss rate in an experimental mouse model of pulmonary fibrosis. Conversely, the selective PLK1 inhibitor GSK461364 showed anti-fibrotic effects without worsening the mortality or weight loss rate.

PLK is a serine/threonine kinase that regulates the cell cycle. It is involved in mitotic initiation and termination, spindle formation, cytokinesis, and meiosis [20][28]. PLK consists of five members: PLK1-5. PLK1 is part of a regulatory network that controls the activity of the Cdk1/cyclin B complex and entry into mitosis at the G2/M transition [20] [29]. PLK1 is highly expressed in embryos as well as in rapidly proliferating adult cells and tissues [30]. Recently, Chen et al. reported that the PLK1 expression was elevated in primary hepatic stellate cells isolated from an experimental mouse model of liver fibrosis. They further reported that blocking PLK1 effectively suppressed liver fibrosis by inhibiting hepatic stellate cell activation [31]. However, the effect of PLK1 inhibition on pulmonary fibrosis has never been investigated. In the present study, we demonstrated that PLK1 expression was upregulated in fibroblasts, PLK1 inhibition suppressed fibroblast proliferation, and PLK1 inhibition ameliorated experimental pulmonary fibrosis in mice. These results suggest that PLK1 is a potential therapeutic target in lung fibrosis.

In contrast to PLK1, PLK2 is a centrosomal kinase expressed primarily in G1 phase [32]. Although inhibition of either PLK1 or PLK2 suppresses cell proliferation, the effect of PLK2 inhibition is not considered to be as strong as that of PLK1 inhibition [33]. Kant et al. reported that genetic deletion of PLK2 in mice induces a spontaneous fibrotic change in lung characterized by alveolar wall thickening and increased collagen deposition [34]. In the present study, we found that PLK2 expression was upregulated in lung epithelial cells and that PLK1/2 inhibition suppressed not only lung fibroblast proliferation but also lung epithelial cell proliferation. These results suggest that the increase in the mortality rate and weight loss by PLK1/2 inhibition in our experimental mouse model of lung fibrosis might have been due to alveolar injury and the inhibition of regeneration of lung epithelial cells.

In the present study, we developed a practical in silico screening approach to identify new therapeutic candidates for pulmonary fibrosis followed by wet-lab validation studies. Although the drug list of L1000CDS2, a drug and small-molecule discovery tool, included both BI2536 and GSK461364, only BI2536 appeared near the top of the list in our systematic computational approach. The present findings suggest that this was due to the lack of an inhibitory effect on pro-fibrotic gene expression by GSK461364. However, BI2536, which was selected for its superior inhibitory effect on pro-fibrotic genes, ameliorated pulmonary fibrosis but also increased the mortality rate, suggesting a limitation of in silico screening, which attempts to find new drugs based solely on their effects on gene expression. However, with further analyses based on the experimental results of BI2536, we were able to select GSK461364 as a promising candidate drug for pulmonary fibrosis. Taken together, our findings suggest that while in silico screening is useful, it is essential to fully determine the biological activities of candidates by wet-lab validation studies.

Another advantage of drug repositioning by in silico screening is the potential identification of new candidates from among known drugs, thus making it easier to predict their side effects and toxicity. Recent studies have suggested that PLK1 is upregulated in various cancers and that PLK1 inhibition has anti-tumor effects [35]. In a phase I study in patients with advanced solid malignancies, GSK461364 was reported to have various side effects, including neutropenia, anemia, and thrombocytopenia [36]. In that study, myelosuppression was observed in patients who received GSK461364 at doses of ≥ 100 mg twice weekly but not in patients who received 25 mg of GSK461364 twice weekly for all cycles [36]. The human equivalent dose (HED), which takes into account the body surface area, is commonly used to calculate the dose that produces equivalent effects in humans to the dose studied in animal experiments. To convert the mouse dose to the HED, the concentration of drugs should be divided by 12.3 [37]. Therefore, if we were to calculate the starting dose of GSK461364 for human studies using the 5 mg/kg mouse dose, the dose would be 0.405 mg/kg for a 60 kg human. A dose of 5 mg/kg twice weekly in mice is considered equivalent to 24.3 mg twice weekly in a 60 kg human, which is almost the same as the minimum dose used in the phase I study. Therefore, in humans, GSK461364 is expected to have well-tolerated side effects at doses that have anti-fibrotic effects.

One limitation of our study is the specificity of BI2536 as an inhibitor of PLKs. BI2536 has been reported to have strong inhibitory activity for not just PLK1/2 but also PLK3 [38]. In this study, although we focused on the differences in the expression of PLK1 and PLK2 and their inhibitory effects for each cell type, PLK3 inhibition by BI2536 might also have affected the present results. Another limitation is that the effects of PLK inhibition on cell types other than fibroblasts and epithelial cells have not fully been investigated. The effect on immune cells may be particularly important. In this study, the effect of BI2536 administration on BALF did not be examined. Therefore. it remains possible that the effect of BI2536 administration on immune cells influenced the results of in vivo experiments.

## Conclusions

In summary, the present study demonstrated the antifibrotic activity of the selective PLK1 inhibitor GSK461364. Although the PLK1/2 inhibitor BI2536 was selected by an in silico analysis, the results of wet-lab validation studies demonstrated the usefulness of GSK461364. Computational approaches leveraging public gene expression data are useful for identifying novel antifibrotics. However, our results indicate that in vivo studies are still essential for investigating the biological effects and usefulness of candidate drugs. The present results may offer new insight into the development of therapies for pulmonary fibrosis.

## Electronic supplementary material

Below is the link to the electronic supplementary material.

Additional file 1: Detailed methods, and figure legends of Figure E1-E6.

Additional file 2: **Figure E1. **BI2536, a PLK1/2 inhibitor, showed a tendency to attenuate pulmonary fibrosis. **Figure E2. **PLK1 expression in alveolar type 2 cells and PLK2 expression in myofibroblast. **Figure E3. **The analysis of bronchoalveolar lavage fluid in mice treated with bleomycin and GSK461364. **Figure E4.** BI2536 downregulates the mRNA expression of *Col1a1*. **Figure E5.** Expanded blots from Figure 6C. **Figure E6.** Expanded blots from Figure 6F.

## Data Availability

All data generated or analysed during this study are included in this published article and its supplementary information files.

## Abbreviations

IPF: Idiopathic pulmonary fibrosis
PLK: Polo-like kinase
DEGs: Differentially expressed genes
GEO: Gene Expression Omnibus
L1000CDS2: L1000 Characteristic Direction Signature Search Engine
Ep-CAM: the epithelial-cell adhesion molecule
FGF-2: Fibroblast growth factor-2
PDGF: Platelet-derived growth factor
H&E: Hematoxylin and eosin
Ashcroft score: A numeric fibrotic scale
BAL: Bronchoalveolar lavage
BALF: BAL fluid
α-SMA: Alpha smooth muscle actin
pro-SPC: Pro-surfactant protein C
Ep-CAM: Epithelial-cell adhesion molecule
HED: Human equivalent dose
