# Supplementary material for: A polo-like kinase inhibitor identified by computational repositioning attenuates pulmonary fibrosis
Source: Respir Res. 2023 Jun 2;24:148. doi: 10.1186/s12931-023-02446-x (PMC10236721; doi:10.1186/s12931-023-02446-x)
Supplement: Supplementary file 1 — Additional file 1: Detailed methods, and figure legends of Figure E1-E6. [file 12931_2023_2446_MOESM1_ESM.docx]

**A polo-like kinase inhibitor identified by computational repositioning attenuates pulmonary fibrosis**

Takeshi Imakura^1^, Seidai Sato^1^, Kazuya Koyama^1^, Hirohisa Ogawa^2^, Takahiro Niimura^3^, Kojin Murakami^1^, Yuya Yamashita^1^, Keiko Haji^1^, Nobuhito Naito^1^, Kozo Kagawa^1^, Hiroshi Kawano^1^, Yoshito Zamami^3 4^, Keisuke Ishizawa^3^, Yasuhiko Nishioka^1^

^1^Department of Respiratory Medicine and Rheumatology, Graduate School of Biomedical Sciences, Tokushima University, Tokushima, Japan

^2^Department of Pathology and Laboratory Medicine, Graduate School of Biomedical Sciences, Tokushima University, Tokushima, Japan

^3^Department of Clinical Pharmacology and Therapeutics, Graduate School of Biomedical Sciences, Tokushima University, Tokushima, Japan

^4^Department of Pharmacy, Okayama University Hospital, Okayama, Japan

**SUPPLEMENTAL DATA**

*Methods*

BI2536 and GSK461364 were purchased from Selleck Biotech (Tokyo, Japan). Bleomycin was purchased from Nippon Kayaku Co. (Tokyo, Japan). Recombinant fibroblast growth factor-2 (FGF-2) was purchased from PeproTech (Rocky Hill, NJ, USA). Recombinant Human platelet-derived growth factor-BB (PDGF-BB) and recombinant Human TGF-beta 1 (TGF-β1) were purchased from R&D Systems, Inc. (Minneapolis, MN, USA). Anti-polo-like kinase (PLK) 1 antibody (PA1-41319; Invitrogen, Carlsbad, CA, USA), anti-PLK1 antibody (ab17056, Abcam, Cambridge, UK), anti-PLK1 antibody (#4513S; Cell Signaling Technology, Danvers, MA, USA), anti-PLK2 antibody (#14812; Cell Signaling Technology), anti-PLK2 antibody (sc-374643, Santa Cruz, Dallas, TX, USA), anti-actin β antibody (sc-47778; Santa Cruz), anti-alpha smooth muscle actin (α-SMA) antibody (ab7817; Abcam), anti-α-SMA antibody (32575; Abcam), anti-pro-surfactant Protein C (pro-SPC) antibody (ab90716; Abcam), monoclonal antibody to Ki-67 (AM33074PU-S; Acris, Herford, Germany), anti-epithelial-cell adhesion molecule (Ep-CAM) antibody biotin conjugate (#13-5791-82; Invitrogen), mouse CD45 microbeads (#130-052-301; Miltenyi Biotec Inc., Auburn, CA, USA), and Streptavidin MicroBeads (#130-048-102; Miltenyi Biotec Inc.) were used.

*In silico analyses*

The gene expression profile used in our analysis was obtained from the Gene Expression Omnibus (GEO) database (https://www.ncbi.nlm.nih.gov/geo/), and we obtained the microarray data of idiopathic pulmonary fibrosis (IPF) in GSE5774. GSE5774 was based on the GPL4255 platform (NIH-NIEHS/Agilent Human Familial IIP 43K array). We used the GEO2R online analysis tool (https://www.ncbi.nlm.nih.gov/geo/geo2r/) to detect the differentially expressed genes (DEGs) between IPF and normal samples. Genes with an adjusted P-value of <0.05 and |log_2_FC| >0.585 were defined as DEGs. We used the L1000 Characteristic Direction Signature Search Engine (L1000CDS2) (http://amp.pharm. mssm.edu/L1000CDS2), a drug and small-molecule discovery tool provided by the NIH, to search for therapeutic candidates for IPF. By inputting DEGs between IPF and normal samples in the L1000CDS2, we searched for therapeutic candidates that counteracted DEGs. The search engine of the L1000CDS2 returns the top 50 matched drugs or small molecules. For the further refinement of candidate drugs or small molecules, the search was conducted again with a more stringent requirement (genes had to meet the adjusted P-value of <0.05 and |log_2_FC| >1).

*Primary culture*

B6 murine lung fibroblasts were primary cultured from lung tissue of C57BL/6 mice. Cells were maintained in Dulbecco's Modified Eagle's medium (DMEM) supplemented with 10% fetal bovine serum (FBS), penicillin (100 U/ml), and streptomycin (50 μg/ml). Cells were cultured at 37 °C in a humidified atmosphere of 5% CO_2_ in air.

*Cell lines*

The murine alveolar epithelial cell lines, LA4 were purchased from the American Type Culture Collection, ATCC (Manassas, VA, USA). Cells were maintained in Dulbecco's Modified Eagle's medium (DMEM) supplemented with 10% FBS, penicillin (100 U/ml), and streptomycin (50 μg/ml). Cells were cultured at 37 °C in a humidified atmosphere of 5% CO_2_ in air.

*Isolation of primary murine alveolar epithelial cells*

Isolation of primary murine alveolar epithelial cells was performed as previously described [E1]. In brief, isolation was performed using negative depletion with CD45 MicroBeads (#130-052-301; Miltenyi Biotec Inc.) and positive selection for Ep-CAM (#13-5791-82; Invitrogen) by magnetic labeling with Streptavidin MicroBeads (#130-048-102; Miltenyi Biotec Inc.) in magnetic cell sorting LS Columns .

*Proliferation assay*

Murine lung fibroblasts (5×10^3^ cells/well) were seeded onto 96-well plates and cultured with various concentration of BI2536 (3-300 nM) or GSK461364 (3-300 nM) in the presence of FGF-2 (30 ng/ml) or PDGF (100 ng/ml) for 48 h. Murine lung epithelial cells (LA4; 5×10^3^ cells/well) were seeded onto 96-well plates and cultured with various concentrations of BI2536 (3-300 nM) or GSK461364 (3-300 nM) in the presence of FGF-2 (30 ng/ml) for 48 h. [^3^H] thymidine deoxyribose (^3^H-TdR) at 1 µCi/well was pulsed for the final 24 h, and the incorporation of ^3^H-TdR was measured using a liquid scintillation counter [E2].

*Immunoblot analyses*

Cell extracts of murine fibroblast cells and alveolar epithelial cells stimulated with FGF-2 (30 ng/ml) (fibroblast cells and epithelial cells) or PDGF (100 ng/ml) (fibroblast cells) were collected after culturing for 24 h in DMEM containing 0.1% FBS. Whole-cell extracts were prepared with M-PER reagents (Thermo Fisher Scientific, Waltham, MA USA) containing phosphatase and protease inhibitor cocktails (Roche, Basel, Switzerland). Protein concentrations were measured using the Bradford method. The same amounts of total cell extract proteins were electrophoresed on 4%-12% NuPAGE Bis-tris Mini Gels and transferred onto polyvinylidene difluoride membranes (Millipore, Billerica, MA) using the WSE-4040 HorizeBLOT 4M-R system (ATTO, Tokyo, Japan). The membrane was treated with the blocking agent Blocking One (Nacalai Tesque) for 1 h and incubated at 4 °C overnight with the first antibodies. Following four-times washes, the membrane was incubated with horseradish peroxidase-conjugated secondary antibodies (1:2000 dilution, GE Healthcare, Fairfield, CT) in buffer at room temperature for one hour. The membrane was developed using Amersham ECL Western Blotting Detection Reagents (GE Healthcare, Fairfield, CT), and signals were detected using an enhanced chemiluminescence system (GE Healthcare, Fairfield, CT) [E3]. The intensity of the bands was quantified using the public domain National Institutes of Health imaging program (W. Rasband, Research Service Branch; National Institutes of Health, Bethesda, MD, USA). The first antibodies used were as follows: anti-PLK1 antibody (1:1000 dilution, #4513S; Cell Signaling Technology), anti-PLK2 antibody (1:1000 dilution, #14812; Cell Signaling Technology), anti-actin β antibody (1:1000 dilution, sc-47778; Santa Cruz).

*Bleomycin-induced pulmonary fibrosis in mice*

Eight-week-old C57BL/6 mice were purchased from Charles River Japan (Kanagawa, Japan). Mice were maintained in the animal facility of Tokushima University under specific pathogen-free conditions according to the guidelines of our university [E4]. The present study was approved by the Institutional Animal Care and Use Committee of Tokushima University (Permission Number: T2020-32).

Mice received a single intra-tracheal instillation of bleomycin (3 mg/kg) on day 0. BI2536 (5 mg/kg, 10 mg/kg or 20 mg/kg) was administered by intraperitoneal instillation twice a week from day 0 until day 21, as was GSK461364 (5 mg/kg). On the day of the experiment, mice were weighed. Lung tissues analyzed on day 21 were used to determine the Ashcroft score (left lung) and for the hydroxyproline colorimetric assay (right lung).

*Histopathology*

Right lung tissues were harvested, fixed in 10% formalin, and embedded in paraffin. Three-micrometer-thick sections were stained with hematoxylin and eosin (H&E) or Azan Mallory. In the quantitative analysis, a numeric fibrotic scale was used (Ashcroft score) [E5]. The mean score was considered to be the fibrotic score.

*Hydroxyproline colorimetric assay*

Hydroxyproline colorimetric assay was performed 21 days after bleomycin instillation using a Bio-vision hydroxyproline assay kit (BioVision, Mount View, CA, USA). Hydroxyproline is an amino acid that is found in collagen, which is the main structural protein in connective tissues. Therefore, the amount of hydroxyproline in a lung tissue can be used as an indicator of the amount of collagen present and is commonly used to monitor the progression of fibrotic lesions characterized by an excessive deposition of collagen.

*Bronchoalveolar lavage*

GSK461364 (5 mg/kg) was administered by intraperitoneal instillation twice a week until day 21 after intratracheal instillation of 3 mg/kg NaCl or bleomycin on day 0. On day 21, Bronchoalveolar lavage (BAL) was performed 5 times with saline (1 mL) using a soft cannula [E6]. After counting the number of cells in the BAL fluid (BALF), cells were cytospun onto glass slides and stained with Diff-Quick (Baxter, Miami, FL, USA) for cell classification.

*Immunofluorescence staining*

After the paraffin-embedded lung sections were deparaffinized, the antigen was activated in an autoclave (120 °C, 15 minutes) with Dako REAL™ Target Retrieval Solution (#S203130; Dako, Glostrup, Denmark). Lung sections were stained with primary antibodies at 4 °C overnight and subsequently stained with fluorescence-conjugated secondary antibodies and 4’,6-diamidino-2-phenylindole at room temperature for 1 h. Fluorescence images were captured with a confocal laser scanning microscope at 200× magnification (Nikon A1R resonant scanning confocal system; Nikon, Tokyo, Japan). The primary antibodies used were as follows: anti-pro-SPC antibody (1:1000 dilution, ab90716; Abcam), anti-α-SMA antibody (1:150 dilution, ab7817; Abcam), anti-PLK1 antibody (1:100 dilution, PA1-41319; Invitrogen), anti-PLK2 antibody (1:50 dilution, sc-374643; Santa Cruz) and monoclonal antibody to Ki-67 (1:200 dilution, AM33074PU-S; Acris).

*Quantitative real-time polymerase chain reaction*

Quantitative real-time polymerase chain reaction (PCR) was performed as previously described [E7]. Total cellular RNA was extracted using am RNeasy Mini Kit (Qiagen, Valencia, CA, USA) and was reverse-transcribed to cDNA using a High Capacity cDNA Reverse Transcription Kit (Applied Biosystems, Carlsbad, CA, USA) according to the manufacturer’s instructions. Reverse-transcription (RT)-PCR was performed using the CFX96 real-time PCR system (Bio-Rad, Hercules, CA, USA) and the SYBR Premix Ex Taq (TAKARA, Kyoto, Japan).

The sequences of primers used were as follows:

*Mouse Plk1* forward, 5’-TGA CGA GTT CTT CAC TTC TGG CTA-3’

*Mouse Plk1* reverse, 5’-ATT GCG GAA ATA GTT GAG GAG AGT-3’

*Mouse Plk2* forward, 5’-GCA GAC ACA GTG GCA AGA GT-3’

*Mouse Plk2* reverse, 5’-CTG GTA CCC AAA GCC GTA TT-3’

*Mouse Col1a1* forward, 5’-TCT GCG ACA ACG GCA AGG TG-3’,

*Mouse Col1a1* reverse, 5’-GAC GCC GGT GGT TTC TTG GT-3’,

*Mouse Acta2* forward, 5’-GAG CGT GGC TAT TCC TTC GT-3’,

*Mouse Acta2* reverse, 5’-GCC CAT CAG GCA ACT CGT AA-3’,

All mRNA data were normalized with 18s ribosomal RNA expression. For this, TaqMan® Universal PCR Master Mix (#4304437; Applied Biosystems, Waltham, MA, USA) and predesigned primer pairs for Rn45s; H (Rn03928990-g1) were used.

*Statistical* *analyses*

The significance of differences was analyzed using unpaired *t-*test for comparisons between two groups or a one-way analysis of variance followed by Dunnett’s test for comparisons between more than two groups. *P* values of <0.05 were considered to indicate statistical significance. These statistical analyses were performed with the GraphPad Prism software program (Ver. 5.01; GraphPad Software Inc., Prism Software, Irvine, CA, USA).

**References**

E1. Messier EM, Mason RJ, Kosmider B. Efficient and rapid isolation and purification of mouse alveolar type II epithelial cells. Exp Lung Res 2012; 38: 363-73.

E2. Aono Y, Nishioka Y, Inayama M, Ugai M, Kishi J, Uehara H, Izumi K, Sone S. Imatinib as a novel antifibrotic agent in bleomycin-induced pulmonary fibrosis in mice. Am J Respir Crit Care Med 2005; 171: 1279-85.

E3.　Sato S, Shinohara S, Hayashi S, Morizumi S, Abe S, Okazaki H, Chen Y, Goto H, Aono Y, Ogawa H, Koyama K, Nishimura H, Kawano H, Toyoda Y, Uehara H, Nishioka Y. Anti-fibrotic efficacy of nintedanib in pulmonary fibrosis via the inhibition of fibrocyte activity. Respir Res 2017; 18: 172.

E4. Nishioka Y, Nishimura N, Suzuki Y, Sone S. Human monocyte-derived and CD83(+) blood dendritic cells enhance NK cell-mediated cytotoxicity. Eur J Immunol 2001; 31: 2633-41.

E5. Ashcroft T, Simpson JM, Timbrell V. Simple method of estimating severity of pulmonary fibrosis on a numerical scale. J Clin Pathol 1988; 41: 467-70.

E6. Aono Y, Kishi M, Yokota Y, Azuma M, Kinoshita K, Takezaki A, Sato S, Kawano H, Kishi J, Goto H, Uehara H, Izumi K, Nishioka Y. Role of platelet-derived growth factor/platelet-derived growth factor receptor axis in the trafficking of circulating fibrocytes in pulmonary fibrosis. Am J Respir Cell Mol Biol. 2014; 51: 793-801.

E7. Chomczynski P, Sacchi N. The single-step method of RNA isolation by acid guanidinium thiocyanate-phenol-chloroform extraction: twenty-something years on. Nat Protoc 2006; 1: 581-5.

**Figure legends**

**Figure E1. BI2536, a PLK1/2 inhibitor, showed a tendency to attenuate pulmonary fibrosis.** C57BL/6 mice received bleomycin (3 mg/kg, intratracheal instillation) on day 0. BI2536 (injected intraperitoneally at a dose of 5, 10 or 20 mg/kg) or vehicle twice a week. Analyses were performed on day 21. (A) A histological examination was performed with H&E staining. Yellow arrows indicate fibrotic region. Scale bars, 1000 mm. (B) The fibrotic changes in the lungs were quantified with a numerical fibrotic score (Ashcroft score) histopathologically (n = 3, control group; n = 6, bleomycin-alone group; n = 6, bleomycin + BI2536 5 mg/kg group; n = 3, bleomycin + BI2536 10 mg/kg group; n = 3, bleomycin + BI2536 20 mg/kg group). Data were analyzed using a one-way analysis of variance followed by Dunnett’s multiple comparison test and were displayed as the mean ± SD. For all graphs: NS = not significant; ** *P* = 0.001-0.01; *** *P* <0.0001. The *P* values of each experiment are shown above each figure.

**Figure E2. PLK1 expression in alveolar type 2 cells and PLK2 expression in myofibroblasts.** C57BL/6 mice received bleomycin (3 mg/kg, intratracheal instillation) on day 0. The lung tissue was harvested on day 21. (A-B) Paraffin-embedded lung sections were stained with an anti-pro-SPC antibody (green) and an anti-PLK1 antibody (red) or with an anti- α-SMA antibody (green) or with and anti-PLK2 antibody (red). Representative images of immunofluorescence staining in control group or bleomycin-treated group are shown. Scale bars, 100 mm. (C-D) C57BL/6 mice received bleomycin (3 mg/kg, intratracheal instillation) on day 0. The lung tissue was harvested on day 21. CD45^-^Ep-CAM^-^ or CD45^+^Ep-CAM^+^ cells were purified. (C) The mRNA expression of *Plk1* in CD45^-^ Ep-CAM^-^ cells or CD45^+^ Ep-CAM^+^. (D) The mRNA expression of *Plk2* in CD45^-^ Ep-CAM^-^ cells or CD45^+^ Ep-CAM^+^. Data were analyzed using paired *t*-test and displayed as a dot plot and the mean ± SD (n = 3 for CD45^-^ Ep-CAM^-^, n = 3 for CD45^-^ Ep-CAM^+^ cells). The *P* values of each experiment are shown above each figure.

**Figure E3. The analysis of bronchoalveolar lavage fluid in mice treated with bleomycin and GSK461364.** C57BL/6 mice received bleomycin (3 mg/kg, intratracheal instillation) on day 0. (A-D) GSK461364 (injected intraperitoneally at a dose of 5 mg/kg) or vehicle twice a week. After mice were killed on day 21, the bronchoalveolar lavage fluid was collected and analyzed (n = 3, control group; n = 5, bleomycin-alone group; n = 7, bleomycin + GSK461364 group). Data were analyzed using a one-way analysis of variance followed by Dunnett’s multiple comparison test and were displayed as the mean ± SD. For all graphs: NS = not significant; ** *P* = 0.001-0.01; *** *P <*0.0001. The *P* values of each experiment are shown above each figure.

**Figure E4. BI2536 downregulates the mRNA expression of *Col1a1*.** (A) Genes suppressed or upregulated by BI2536 were extracted from the L1000 Characteristic Direction Signature Search Engine. (B-C) The mRNA expression of *Col1a1 or Acta2* in cultured murine primary fibroblasts stimulated with 10 ng/ml of TGF-β1 or 48 h was analyzed by quantitative PCR. The murine primary fibroblasts cultured with BI2536 3 nM or 30 nM (n = 3, control group; n = 3, TGF-β1 group; n = 3, TGF-β1 + BI2536 3 nM group; n = 3, TGF-β1 + BI2536 30 nM group). (D-E) The mRNA expression of *Col1a1 or Acta2* in cultured murine primary fibroblasts stimulated with TGF-β1 10 ng/ml for 48 h was analyzed by quantitative PCR. The murine primary fibroblasts cultured with GSK461364 3 nM or 30 nM (n = 3, control group; n = 3, TGF-β1 group; n = 3, TGF-β1 GSK461364 3 nM group; n = 3, TGF-β1 + GSK461364 30 nM group). Data were analyzed using a one-way analysis of variance followed by Dunnett’s multiple comparison test and displayed as the mean ± SD. For all graphs: NS = not significant; * *P =* 0.01-0.05; ** *P* = 0.001-0.01; *** *P* <0.0001. The *P* values of each experiment are shown above each figure.

**Figure E5. Expanded blots from Figure 6C.**

**Figure E6. Expanded blots from Figure 6F.**
