# Supplementary material for: A polo-like kinase inhibitor identified by computational repositioning attenuates pulmonary fibrosis
Source: Respir Res. 2023 Jun 2;24:148. doi: 10.1186/s12931-023-02446-x (PMC10236721; doi:10.1186/s12931-023-02446-x)
Supplement: Supplementary file 2 — Additional file 2: Figure E1. BI2536, a PLK1/2 inhibitor, showed a tendency to attenuate pulmonary fibrosis. Figure E2. PLK1 expression in alveolar type 2 cells and PLK2 expression in myofibroblast. Figure E3. The analysis of bronchoalveolar lavage fluid in mice treated with bleomycin and GSK461364. Figure E4. BI2536 downregulates the mRNA expression of Col1a1. Figure E5. Expanded blots from Figure 6C. Figure E6. Expanded blots from Figure 6F. [file 12931_2023_2446_MOESM2_ESM.pdf]

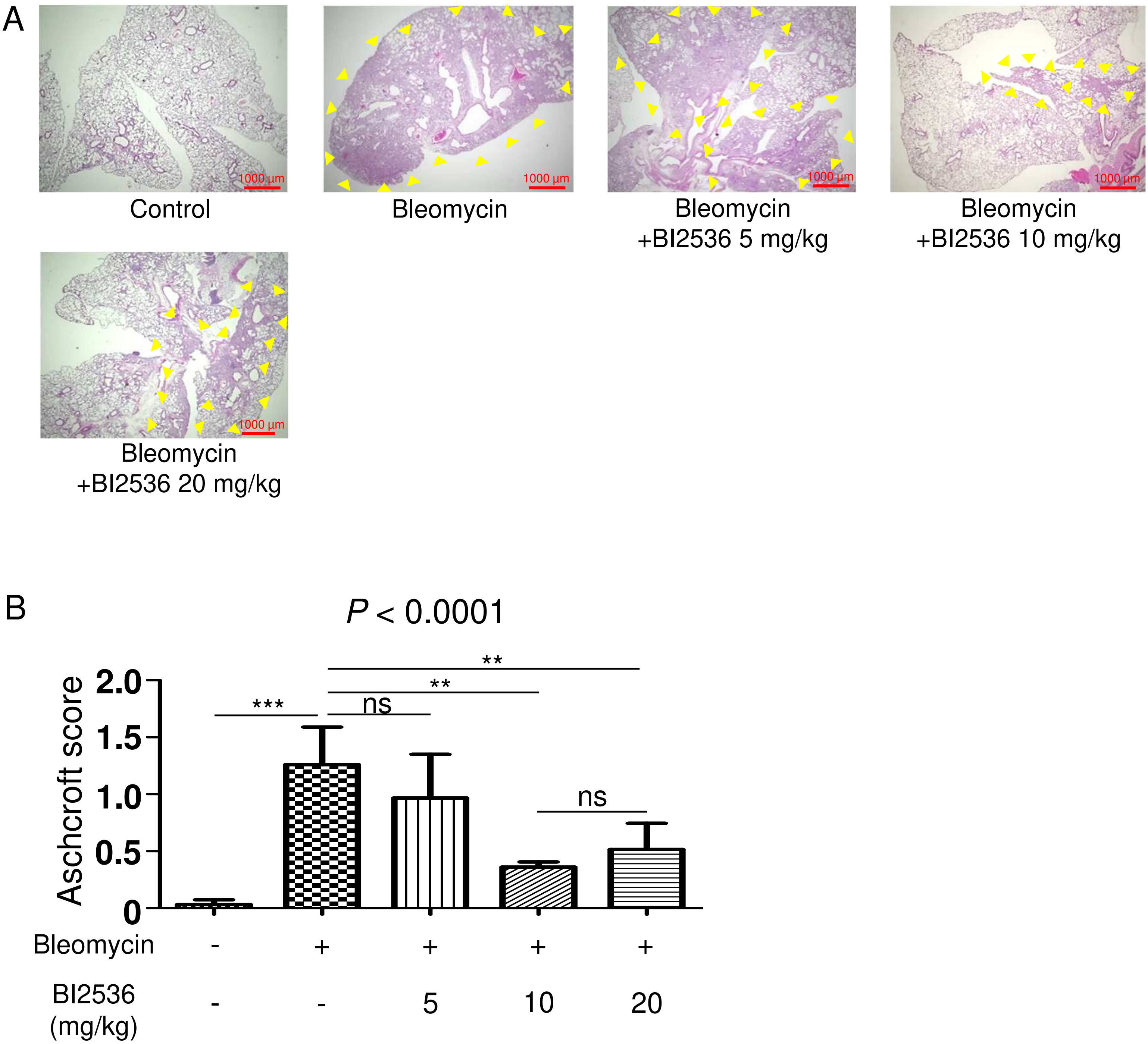
A
1000 µm
Control
Bleomycin
Bleomycin
+BI2536 5 mg/kg
Bleomycin
+BI2536 10 mg/kg
Bleomycin
+BI2536 20 mg/kg
B
P < 0.0001
Aschcroft score
2.0
1.5
1.0
0.5
0
***
ns
**
**
ns
Bleomycin - + + + +
BI2536 (mg/kg) - - 5 10 20

Figure E1

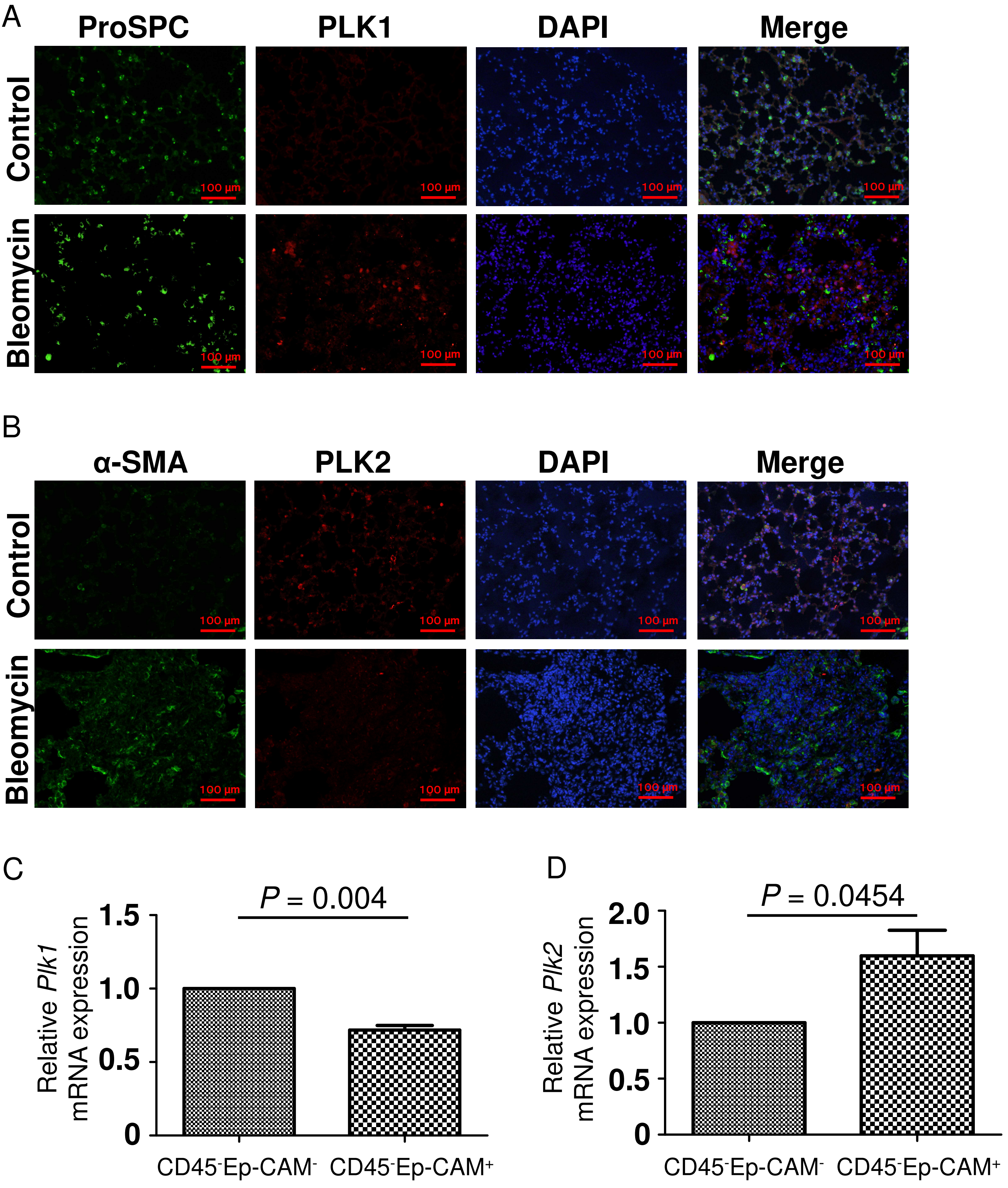
A
ProSPC
PLK1
DAPI
Merge
Control
Bleomycin
100 µm
B
α-SMA
PLK2
DAPI
Merge
Control
Bleomycin
100 µm
C
P = 0.004
Relative Plk1 mRNA expression
1.5
1.0
0.5
0
CD45-Ep-CAM-
CD45-Ep-CAM+
D
P = 0.0454
Relative Plk2 mRNA expression
2.0
1.5
1.0
0.5
0
CD45-Ep-CAM-
CD45-Ep-CAM+

Figure E2

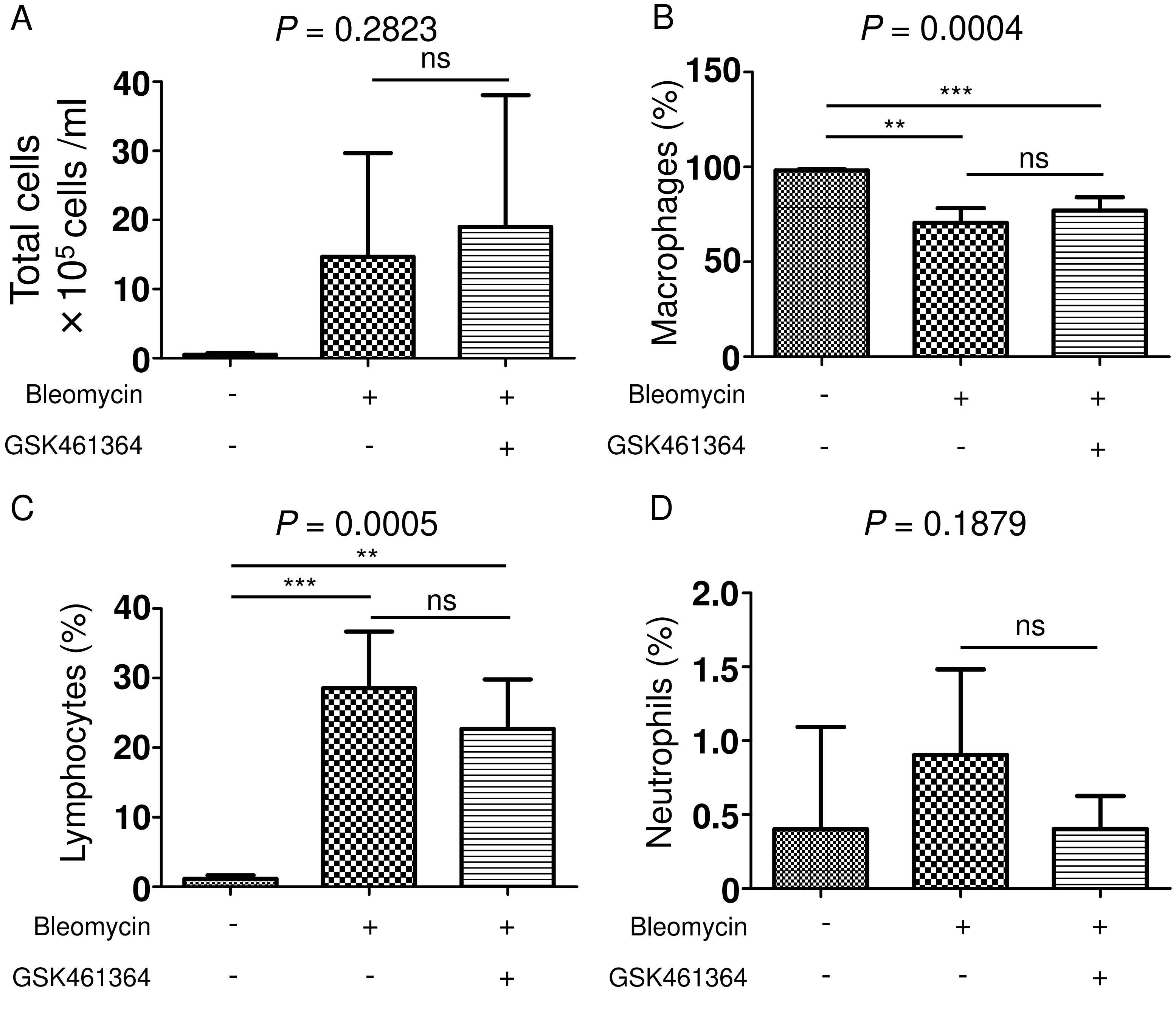
A
P = 0.2823
ns
Total cells × 10⁵ cells /ml
40
30
20
10
0
Bleomycin - + +
GSK461364 - - +
B
P = 0.0004
***
**
ns
Macrophages (%)
150
100
50
0
Bleomycin - + +
GSK461364 - - +
C
P = 0.0005
**
***
ns
Lymphocytes (%)
40
30
20
10
0
Bleomycin - + +
GSK461364 - - +
D
P = 0.1879
ns
Neutrophils (%)
2.0
1.5
1.0
0.5
0
Bleomycin - + +
GSK461364 - - +

Figure E3

A

**Genes suppressed by BI2536**	*CCL2, COL1A1, COL1A2, COL3A1, COL5A1, COL5A2, COL6A1, CXCL12, CXCL14, DMD, EIF4B, FHL2, HOXB2, IFITM1, KRT17, LY6D, S100A2, SFRP1, VCAM1*
**Genes upregulated by BI2536**	*C4BPA, CACNA2D2, CAMK2N1, CD36, CYP51A1, DHCR24, EPAS1, ETV5, FADS1, FDPS, HMOX1, INSIG1, LAMP3, LDLR, LSS, MLPH, PCDH9, SCD, SFTPC, TMEM41B*

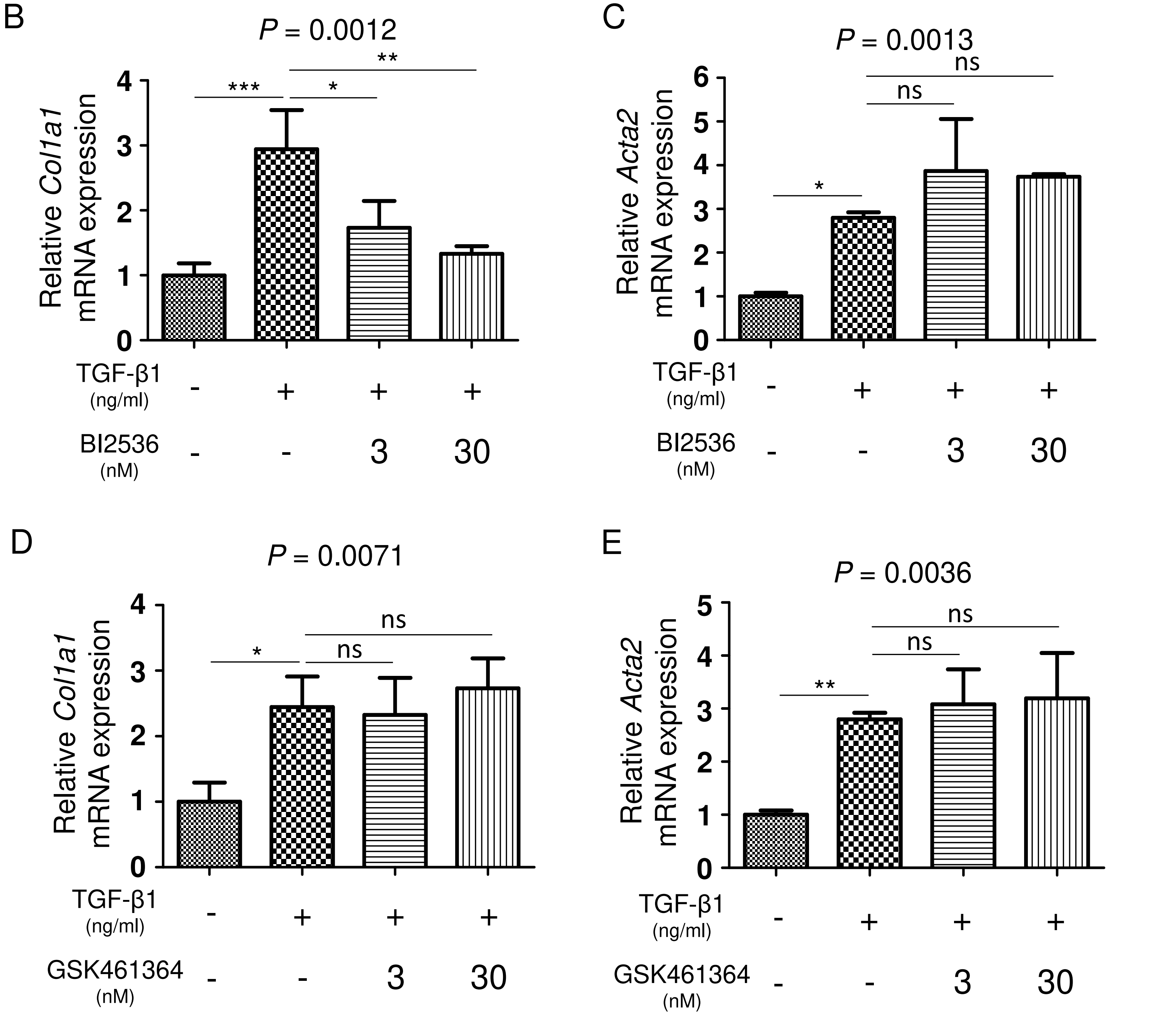

Figure E4

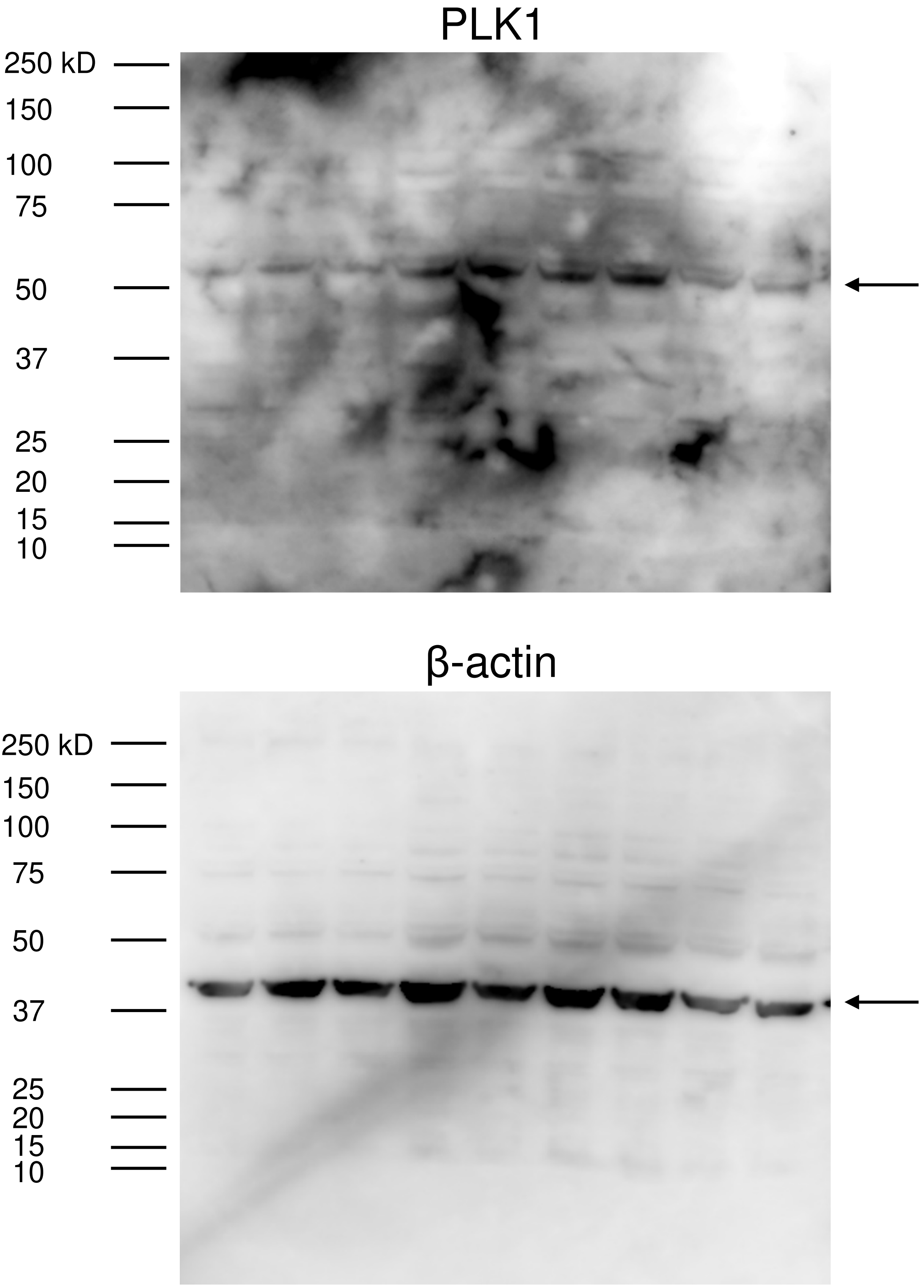
PLK1
250 kD
150
100
75
50
37
25
20
15
10
β-actin
250 kD
150
100
75
50
37
25
20
15
10

Figure E5

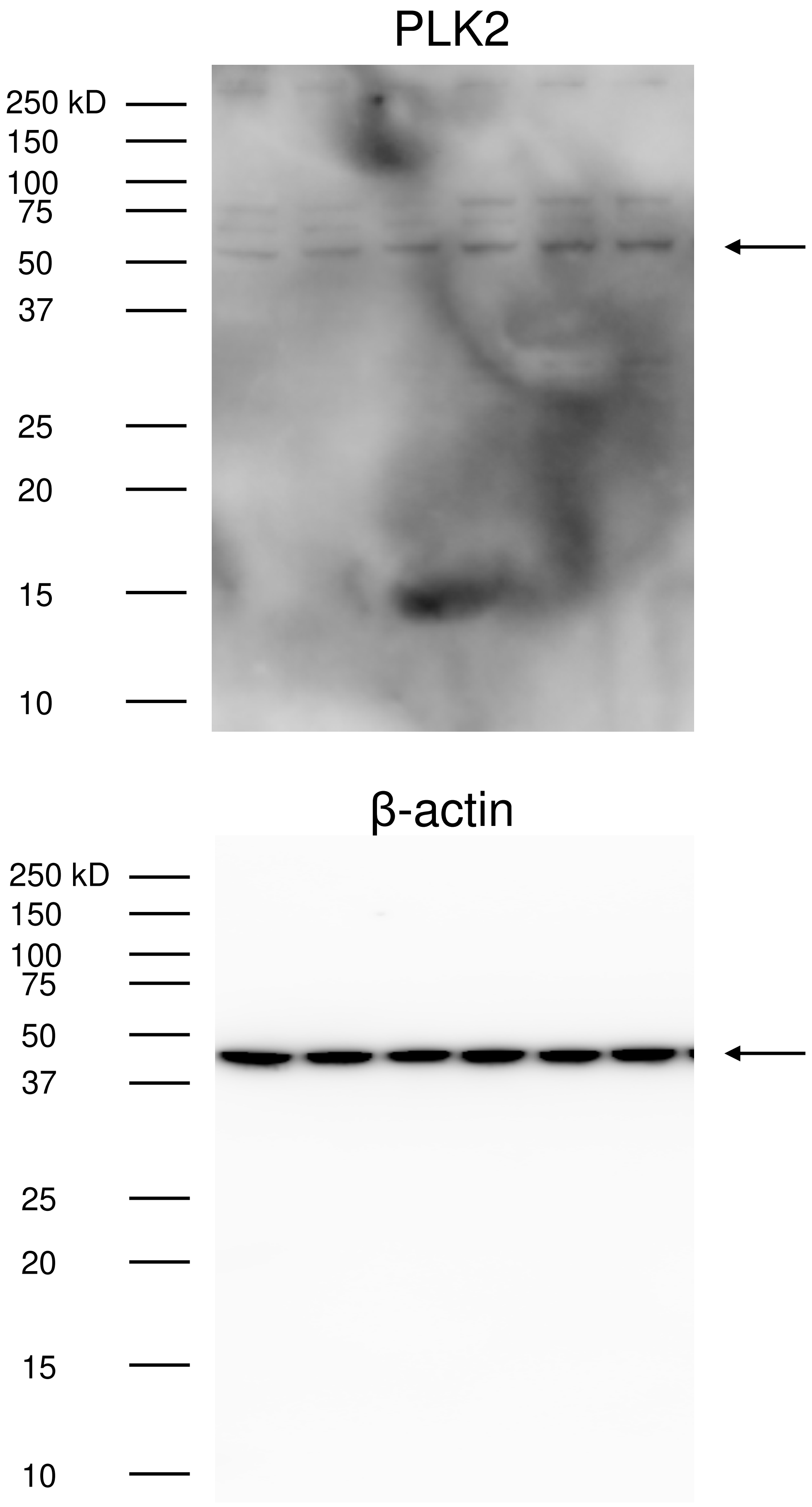
PLK2
250 kD
150
100
75
50
37
25
20
15
10
β-actin
250 kD
150
100
75
50
37
25
20
15
10

Figure E6
